# The antigenic anatomy of SARS-CoV-2 receptor binding domain

**DOI:** 10.1016/j.cell.2021.02.032

**Published:** 2021-04-15

**Authors:** Wanwisa Dejnirattisai, Daming Zhou, Helen M. Ginn, Helen M.E. Duyvesteyn, Piyada Supasa, James Brett Case, Yuguang Zhao, Thomas S. Walter, Alexander J. Mentzer, Chang Liu, Beibei Wang, Guido C. Paesen, Jose Slon-Campos, César López-Camacho, Natasha M. Kafai, Adam L. Bailey, Rita E. Chen, Baoling Ying, Craig Thompson, Jai Bolton, Alex Fyfe, Sunetra Gupta, Tiong Kit Tan, Javier Gilbert-Jaramillo, William James, Michael Knight, Miles W. Carroll, Donal Skelly, Christina Dold, Yanchun Peng, Robert Levin, Tao Dong, Andrew J. Pollard, Julian C. Knight, Paul Klenerman, Nigel Temperton, David R. Hall, Mark A. Williams, Neil G. Paterson, Felicity K.R. Bertram, C. Alistair Siebert, Daniel K. Clare, Andrew Howe, Julika Radecke, Yun Song, Alain R. Townsend, Kuan-Ying A. Huang, Elizabeth E. Fry, Juthathip Mongkolsapaya, Michael S. Diamond, Jingshan Ren, David I. Stuart, Gavin R. Screaton

**Affiliations:** 1 Wellcome Centre for Human Genetics, Nuffield Department of Medicine, University of Oxford, Oxford OX3 7BN, UK; 2 Division of Structural Biology, Nuffield Department of Medicine, University of Oxford, The Wellcome Centre for Human Genetics, Headington, Oxford OX3 7BN, UK; 3 Department of Medicine, Washington University School of Medicine, St. Louis, St. Louis, MO 63110, USA; 4 Oxford University Hospitals NHS Foundation Trust, Oxford, UK; 5 Chinese Academy of Medical Science (CAMS) Oxford Institute (COI), University of Oxford, Oxford OX3 7FZ, UK; 6 Department of Pathology and Immunology, Washington University School of Medicine, St. Louis, St. Louis, MO 63110, USA; 7 Peter Medawar Building for Pathogen Research, Oxford OX1 3SY, UK; 8 Department of Zoology, University of Oxford, Oxford OX1 3SZ, UK; 9 MRC Human Immunology Unit, MRC Weatherall Institute of Molecular Medicine, Radcliffe Department of Medicine, University of Oxford, Oxford OX3 9DS, UK; 10 Sir William Dunn School of Pathology, University of Oxford, Oxford OX1 3RE, UK; 11 National Infection Service, Public Health England (PHE), Porton Down, Salisbury SP4 0JG, UK; 12 Nuffield Department of Clinical Neurosciences, University of Oxford, Oxford OX3 9DU, UK; 13 Department of Paediatrics, Oxford Vaccine Group, University of Oxford, Oxford OX3 7LE, UK; 14 NIHR Oxford Biomedical Research Centre, Oxford OX3 9DU, UK; 15 Worthing Hospital, Worthing BN11 2DH, UK; 16 Nuffield Department of Medicine, University of Oxford, Oxford OX3 7FZ, UK; 17 Viral Pseudotype Unit, Medway School of Pharmacy, University of Kent, Chatham ME4 4TB, UK; 18 Diamond Light Source Ltd, Harwell Science & Innovation Campus, Didcot OX11 0DE, UK; 19 Research Center for Emerging Viral Infections, College of Medicine, Chang Gung University, Taoyuan, Taiwan; 20 Division of Pediatric Infectious Diseases, Department of Pediatrics, Chang Gung Memorial Hospital, Taoyuan, Taiwan; 21 Siriraj Center of Research Excellence in Dengue & Emerging Pathogens, Dean Office for Research, Faculty of Medicine Siriraj Hospital, Mahidol University, Thailand; 22 Department of Molecular Microbiology, Washington University School of Medicine, St. Louis, St. Louis, MO 63110, USA; 23 The Andrew M. and Jane M. Bursky Center for Human Immunology and Immunotherapy Programs, Washington University School of Medicine, St. Louis, St. Louis, MO 63110 USA; 24 Instruct-ERIC, Oxford House, Parkway Court, John Smith Drive, Oxford OX4 2JY, UK

**Keywords:** coronavirus, SARS-CoV-2, anti-RBD antibody, receptor binding domain, antibody, immune responses, virus structure

## Abstract

Antibodies are crucial to immune protection against SARS-CoV-2, with some in emergency use as therapeutics. Here, we identify 377 human monoclonal antibodies (mAbs) recognizing the virus spike and focus mainly on 80 that bind the receptor binding domain (RBD). We devise a competition data-driven method to map RBD binding sites. We find that although antibody binding sites are widely dispersed, neutralizing antibody binding is focused, with nearly all highly inhibitory mAbs (IC_50_ < 0.1 μg/mL) blocking receptor interaction, except for one that binds a unique epitope in the N-terminal domain. Many of these neutralizing mAbs use public V-genes and are close to germline. We dissect the structural basis of recognition for this large panel of antibodies through X-ray crystallography and cryoelectron microscopy of 19 Fab-antigen structures. We find novel binding modes for some potently inhibitory antibodies and demonstrate that strongly neutralizing mAbs protect, prophylactically or therapeutically, in animal models.

## Highlights

• Map 377 mAbs: 19 of 80 recognizing the RBD are potent neutralizers; 1 potent NTD binder
• 19 Fab-antigen complex structures; 80 mAbs mapped on RBD and clustered into 5 epitopes
• Most potent mAbs are ACE2 blockers, neutralize with few ACE2s, some Fabs glycosylated
• mAbs reveal unique examples of NTD binding, RBD binding mode, and LC optimization

## Graphical abstract

**Figure:**
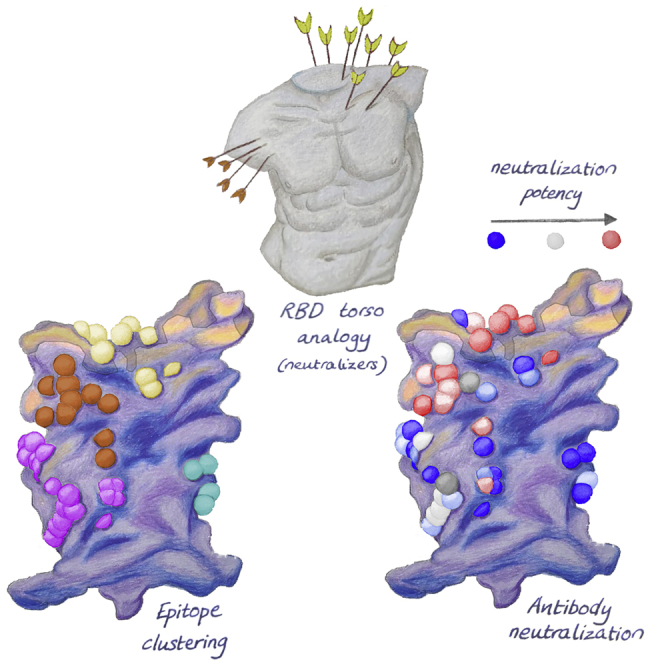

## Teaser

Dejnirattisai et al. present an in-depth study of the human antibody response to SARS-CoV-2 infection. By characterizing 377 human mAbs from recovered COVID-19 patients, and determining 19 protein structures, they construct a map of antibody footprints on the RBD that describes in great detail its antigenic anatomy.

## Introduction

A severe viral acute respiratory syndrome named COVID-19 was first reported in Wuhan, China in December 2019. The virus rapidly disseminated globally leading to the pandemic we are suffering, with over 100 million confirmed infections and over 2.2 million deaths (https://www.worldometers.info/coronavirus/). The causative agent, SARS-CoV-2, is a beta coronavirus, related to SARS-CoV-1 and MERS coronaviruses, which both cause severe respiratory syndromes.

The sequence of SARS-CoV-2 was released in early January 2020 and this led to the mobilization of an unprecedented international scientific response (Chen et al., 2020a). Over 200 vaccine candidates are in development (Krammer, 2020) and 13 are in phase III clinical trials (https://www.who.int/publications/m/item/draft-landscape-of-covid-19-candidate-vaccines) with Novovax and Janssen having reported efficacy recently and Pfizer/BioNTech, Moderna and Oxford-AstraZeneca having received, emergency use authorization (EUA) in a number of countries.

Coronaviruses have 4 structural proteins, nucleocapsid, envelope, membrane, and spike (S). S from both SARS-CoV-2 and SARS-CoV-1 uses angiotensin-converting enzyme 2 (ACE2) as the cell surface receptor (Hoffmann et al., 2020, Li, 2015), ACE2 is expressed in a number of tissues, including epithelial cells of the upper and lower respiratory tracts. S consists of two subunits, S1 that mediates receptor binding and S2 responsible for viral and host cell membrane fusion (Walls et al., 2020; Wrapp et al., 2020). It is a dynamic structure capable of transitioning to a post-fusion state (Cai et al., 2020) by cleavage between S1 and S2 following receptor binding or trypsin treatment. In most SARS-CoV-2 sequences, a furin protease cleavage site is inserted between the S1 and S2 subunits, and mutation of the cleavage site attenuates disease in animal models (Johnson et al., 2020). The S1 fragment, at the membrane distal tip of S, includes an N-terminal domain (NTD) and receptor binding domain (RBD). Although both regions are immunogenic, the RBD contains the interacting surface for ACE2 binding (Lan et al., 2020). Although usually packed down against the top of S2, RBDs can swing upward to engage ACE2 (Roy et al., 2020). Monoclonal antibodies (mAbs) recognize one or both of “up” and “down” conformations (Zhou et al., 2020; Liu et al., 2020). The S protein is relatively conserved between SARS-CoV-2 and SARS-CoV-1 (76%), but the RBD and NTD are less conserved (74% and 50%, respectively) than the S2 domain (90%) (Jaimes et al., 2020). Conservation with MERS-CoV and the seasonal human coronaviruses is much lower (19%–21%). Overall, SARS-CoV-2 antibodies show limited cross-reactivity even with SARS-CoV-1 (Tian et al., 2020).

Previous studies of SARS-CoV-2 have indicated that most potent mAbs bind close to the ACE2 interacting surface on the RBD to block the interaction with ACE2 (Zost et al., 2020; Liu et al., 2020) expressed on target cells or disrupt the pre-fusion conformation (Huo et al., 2020; Yuan et al., 2020a; Zhou et al., 2020). There has been intense interest in S for the development of protective SARS-CoV-2 vaccines or for therapeutic mAbs, several of which are in clinical evaluation and even being deployed under EUA (DeFrancesco, 2020).

Here, we characterize a panel of 377 human mAbs from recovered COVID-19 patients. We devise a generally applicable method combining biophysical competition measurements with a smaller number of crystallographic structure determinations, to pinpoint the attachment site for all 80 mAbs that bind the RBD. The resulting map shows that the antibody footprints cover the majority of the RBD surface, grouping into five epitopes by cluster analysis. In addition, we have determined 19 structures, mainly of Fab fragments with either spike or RBD, by X-ray crystallography or cryoelectron microscopy (cryo-EM). These include many of the most potently inhibitory antibodies, all RBD-binders except for a single N-terminal domain binder. We analyze the modes of binding for antibodies with several public heavy-chain (HC) V-regions. Of these, some engage identical sites through conserved HC CDR1 and CDR2 (H1, H2) interactions, whereas others use variable length HC CDR3s (H3) to bind at different points. We find that shuffling the light-chain pairing within one of these families leads to tighter binding. Other potently neutralizing antibodies have novel interaction sites, and several of these bear somatic mutations that create N-linked glycosylation sites in H1–H3 (Zhang et al., 2016). By studying the valency of antibody binding to virus particles, we show that some of the most potent antibodies can neutralize at low receptor occupancies. The most potent mAbs neutralize the virus in the low picomolar range and show both prophylactic and therapeutic activity in a stringent murine model of SARS-CoV-2 pathogenesis.

## Results

### Characterization of mAbs

**Figure S1:**
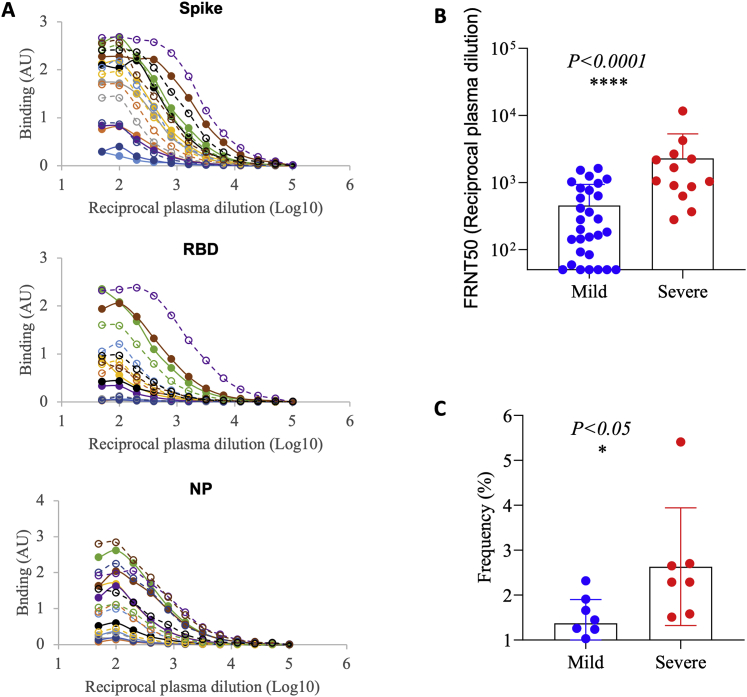
SARS-CoV-2 elicits binding and neutralizing antibodies against trimeric spike, RBD, and NP proteins, related to Figure 1 (A) Plasma from donors with confirmed SARS-CoV-2 infection were collected at 1-2 months after onset of symptoms and tested for binding to SARS-CoV-2 spike, RBD and N proteins by capture ELISA. (B) Neutralizing titers to authentic live virus. Data are representative of one experiment with 42 samples and presented as means ± s.e.m. (C) Comparison of the frequency of spike-reactive IgG expressing B cells in mild cases and severe cases measured by FACS. Small horizontal lines indicate the median. Data are representative of one experiment with 16 samples. The Mann–Whitney U test was used for the analysis and two-tailed P values were calculated (in B and C).

We studied a cohort of 42 patients who had proven SARS-CoV-2 infection diagnosed by qRT-PCR (Table S1). Patients were recruited using the ISARIC protocol following informed consent and recalled following convalescence (31–62 days). ELISAs were performed against full-length stabilized S protein (Wuhan-Hu-1 strain, MN908947) where residues 986 and 987 in the linker between two helices in S2 were mutated to a Pro-Pro sequence to prevent the conversion to the post-fusion helical conformation (Walls et al., 2020; Wrapp et al., 2020), RBD (aa 330–532), or N protein (Figure S1A). As has been described previously, antibody titers varied between patients, and there was a strong correlation between neutralization titer or the level of anti-S expressing memory B cells with disease severity (Chen et al., 2020b) (Figures S1B and S1C).

**Figure S2:**
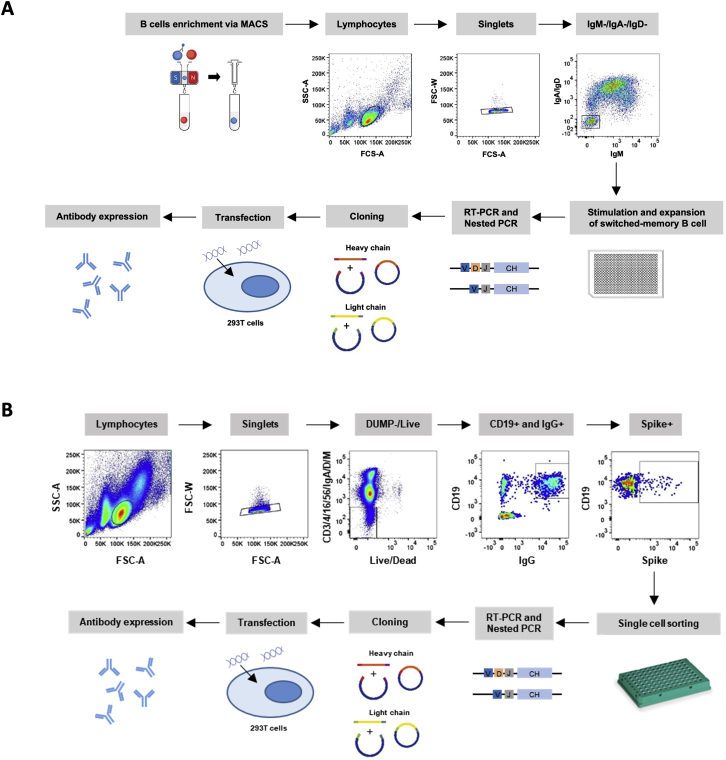
SARS-CoV-2 antibody isolation strategies, related to Figure 1 Human monoclonal antibodies from memory B cells were generated using two different strategies. (A) IgG expressing B cells were isolated and cultured with IL-2, IL-21 and 3T3-msCD40L cells for 13-14 days. Supernatants were harvested and tested for reactivity to spike protein by ELISA. (B) Antigen-specific single B cells were isolated using labeled recombinant spike or RBD proteins as baits. The IgG heavy and light chain variable genes from both strategies were amplified by nested PCR and cloned into expression vectors to produce full-length IgG1 antibodies.

To generate mAbs, two strategies were used. First, immunoglobulin G (IgG)-expressing B cells were sorted, 4 cells per well, cultured with interleukin (IL)-2, IL-21, and 3T3-msCD40L cells for 13–14 days, and supernatants were tested for reactivity to S protein; positive clones were identified by RT-PCR (Figure S2A). In a second method, B cells were stained with labeled S or RBD proteins, and single positive cells were sorted and subjected to RT-PCR (Figure S2B). Cell recovery was higher in the severe COVID-19 cases (Figure S1C), and in total, we isolated mAbs from 16 patients (9 mild, 7 severe).

**Figure S3:**
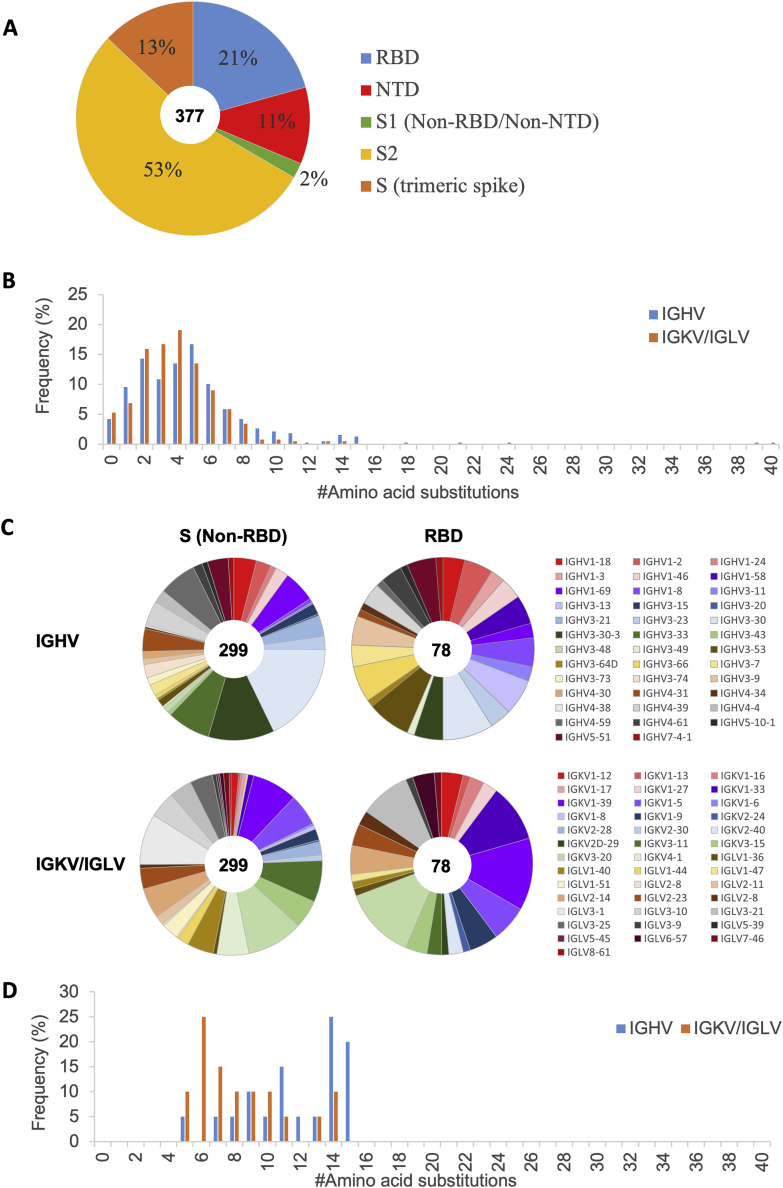
Specificity and sequence analysis of 377 human antibodies, related to Figures 1, 2, and 5 (A) Epitope mapping of SARS-CoV-2 -specific antibodies against the RBD, S1 subunit (aa 16–685) and S2 subunit (aa 686-1213) were evaluated by ELISA, and the NTD-binders were identified by cell-based fluorescent immunoassay. Antibodies interacting with none of the subdomains were defined as trimeric spike. The number in the centers indicate the total number of tested antibodies. (B) Frequency of amino acid substitutions from germline in SARS-CoV2-specific heavy and light chains (n = 377). (C) Repertoire analysis of antibody heavy and light chains of anti-S (Non-RBD) and anti-RBD antibodies. At the center is the number of antibodies. Each slice represents a distinct clone and is proportional to the clone size. (D) Frequency of amino acid substitutions from germline in heavy and light chains of antibodies cross-reacting between SARS-CoV-2 and the 4 seasonal coronaviruses (n = 20).

**Figure 1:**
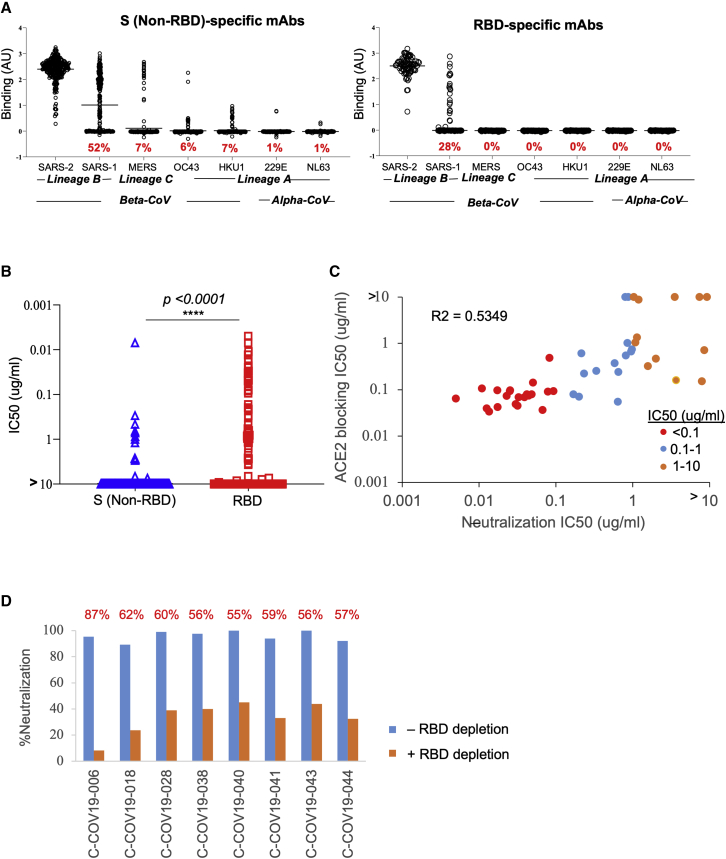
Characterization of SARS-CoV-2-specific mAbs (A) Cross-reactivity of 299 anti-spike (non-RBD) and 78 anti-RBD antibodies to trimeric spike of human alpha- and beta-coronaviruses by capture ELISA. (B) Comparison of neutralization potencies (IC_50_) between anti-spike (non-RBD) and anti-RBD antibodies against authentic SARS-CoV-2 using focus reduction neutralization test (FRNT). The Mann-Whitney U test was used for the analysis and two-tailed p values were calculated. (C) Correlation between SARS-CoV-2 neutralization and RBD:ACE2 blocking by anti-RBD antibodies. Antibodies with IC_50_ <0.1 μg/mL, 0.1–1 μg/mL, and 1–10 μg/mL are highlighted in red, blue, and orange, respectively. (D) Plasma was depleted of RBD-specific antibodies using Ni-NTA beads coated with or without RBD, then evaluated for SARS-CoV-2 neutralizing activity by FRNT assay (n = 8). Results are expressed as percent neutralization of control without plasma. The percentage of depletion of neutralizing antibodies for each sample tested is indicated at the top of each panel. See also Figures S1, S2, and S3.

377 antibodies were produced, which reacted to full-length S by ELISA. mAbs were further screened for reactivity to S1 (34%), S2 (53%), RBD (21%), and the NTD (11%), with the remaining 13% reactive only to full-length trimeric spike (Figure S3A). Analysis of antibody sequences revealed low levels of somatic mutation of germline sequences for both heavy (mean 4.11 ± 2.75 amino acids) and light chains (mean 4.10 ± 2.84 amino acids) (Figure S3B). In general, responses within and between individuals were highly polyclonal with diverse V-gene usage (Figure S3C). We tested cross-reactivity of the 377 anti-S antibodies generated from SARS-CoV-2 patients to full-length S proteins from all human alpha- and beta-coronaviruses (Figure 1A). Cross-reactivity was observed with SARS-CoV-1 (52%), MERS (7%), OC43 (6%), HKU1 (7%), 229E (1%), and NL63 (1%). However, for antibodies recognizing RBD, cross-reactivity was restricted to SARS-CoV-1, the RBD of which shares 74% sequence identity with SARS-CoV-2, much more than the other human CoVs (19%–21%). Antibodies cross-reacting between the RBDs of SARS-CoV-2 and SARS-CoV-1 showed similarly low levels of germline mutation to the whole pool of S reactive antibodies. However, for antibodies cross-reacting between SARS-CoV-2 and the four seasonal coronaviruses, there were more germline mutations, particularly in the heavy chain (Figure S3D). One plausible explanation for the increase in germline mutation in the cross-reactive clones is that they were selected from the memory pool of seasonal coronavirus-specific B cells, rather than generated *de novo* after SARS-CoV-2 infection.

### Neutralization activity of SARS-CoV-2 mAbs

Next, we investigated the neutralizing activity of all 377 mAbs using a focus reduction neutralization test (FRNT) using Vero cells. Only 5% of non-RBD mAbs showed neutralizing activity (IC_50_ <10 μg/mL), whereas 60% of RBD-specific mAbs showed neutralizing activity (Figure 1B) consistent with previous studies of SARS-CoV-1 and SARS-CoV-2 (Barnes et al., 2020).

In total, 19 of 80 anti-RBD antibodies yielded IC_50_ levels of <0.1 μg/mL (Figure 1C), which we define as potent neutralizers. FRNT50 values for a selection of antibodies are shown in Table S2. A number of antibodies outside the RBD had weak neutralizing activity (IC_50_ values of 0.29–7.38 μg/mL). mAb 159, which binds to the NTD (see below), was one of the most potent inhibitory antibodies we obtained with an IC_50_ of 5 ng/mL.

We measured the ability of anti-RBD mAbs to block interaction with ACE2 using a competitive ELISA. For antibodies showing neutralization, there was broad correlation between inhibitory potency and ACE2 blocking, whereas NTD-binding mAb 159 did not block ACE2 binding (Figure 1C).

To investigate the contribution of RBD binding antibodies to neutralization in polyclonal serum, we immunodepleted sera from 8 convalescent donors with recombinant RBD; depletion of anti-RBD activity was confirmed by ELISA. Neutralization assays were performed in RBD-depleted and mock-depleted samples and showed the major contribution made by anti-RBD antibodies with 55%–87% (mean 61.5%) of neutralization due to RBD binders. Although some RBD epitopes (e.g., quaternary epitopes) may be resilient to RBD depletion, this indicates that although the large majority of non-RBD antibodies do not neutralize, those that do have a substantive role in the polyclonal neutralizing response to SARS CoV-2 (Figure 1D).

### Mapping the RBD antigenic surface

To acquire greater insight as to the mAb binding sites on the RBD, we measured pairwise competition between antibodies using biolayer interferometry (BLI) in a 96-well plate format. For 80 antibodies, 4,404 of the 6,340 non-diagonal elements of the square competition matrix were populated. The antibodies were classified into mutually competing groups using cluster analysis (STAR Methods). We derived the topography of binding for all the tested antibodies directly from the competition data with the aid of existing structural data. We expanded the competition matrix to include 3 additional (“external”) antibodies of known binding positions (STAR Methods). The external antibodies and one structure determined in the present study were set to their known positions on a smoothened mesh derived from the solvent-accessible surface of the RBD. The remaining 79 antibodies were assigned randomly to a starting vertex on the mesh and their positions refined by iterative minimization of a simple target function to match observed competition (antibodies were modeled as competing spheres of 22 Å diameter, see STAR Methods). Minimization was performed 1,000 times using Monte Carlo sampling from random starting positions. The results with lowest residuals were filtered using *cluster4x* (Ginn, 2020). The final positions of the mAbs (Table S3) were taken as the sampled position with the lowest average square distance to all other sampled positions. This consensus prediction replicates well the observed competition data (correlation coefficient 0.84). To assess the accuracy of the method, six antibodies whose positions we have since determined (see below), were compared with their predicted locations. The average error was 7.6 Å.

**Figure 2:**
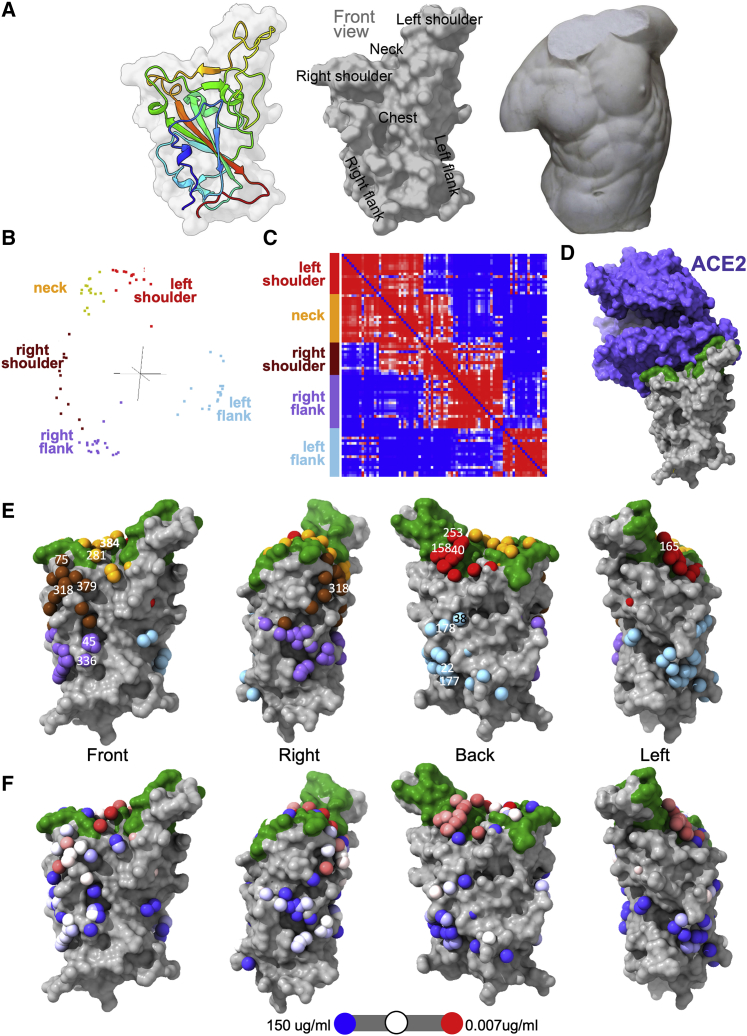
RBD anatomy and epitope definition based on mapping results (A) Pale gray RBD surface with cartoon depiction of one monomer rainbow colored from blue (N terminus) to red (C terminus) alongside gray surface depiction of RBD labeled to correspond to the adjacent torso (Torso Gaddi, Wikipedia, CC BY-SA 3.0, modified in Adobe Photoshop) used by analogy to enable definition of epitopes. (B) Cluster maps showing the output of the mapping algorithm with each spot corresponding to a “located” antibody and color-coded according to epitope. (C) BLI antibody data competition matrix (calculated values) output from cluster analysis showing the clustering into 5 epitopes. (D) RBD (gray)-ACE2 (purple) complex (PDB: 6M0J, (Lan et al., 2020)). RBD residues contacting ACE2 are shown in green. (E) Located antibodies mapped onto the RBD shown as a gray surface with the ACE2-binding site in green. The individual antibodies are depicted as spheres and color coded as in (B), those central to this paper are labeled. (F) As for (E), but antibodies are color-coded according to their ability to neutralize. See inset scale: red, strongest neutralizers; blue, weakest neutralizers. See also Figure S3.

To facilitate interpretation of the results, we introduce a naming convention for the RBD by comparison with a human torso (Figure 2A). The predicted locations, covering most of the RBD surface, were classified into 5 groups using a clustering algorithm (STAR Methods and *cluster4x*) (Ginn, 2020) (Figures 2B and 2C). The left flank cluster is distinct from the other 4 clusters which show marked competition at their boundaries and interact sequentially from the left shoulder, neck, right shoulder to right flank. Competition was strongest between the left shoulder and neck, although the neck and right shoulder groups also cross-compete strongly (Figure 2C).

The ACE2 binding site is shown in Figure 2D, and the positions of the 80 individual antibodies (plus externals) are depicted in Figure 2E. The neck cluster is the site of attachment of a number of antibodies possessing the public IGVH3-53 V-region (Yuan et al., 2020b) and strongly overlaps the ACE2 binding site (Figures 2D and 2E). The left flank cluster includes previously determined structures EY6A, CR3022 and H014, all of which are reported to show neutralizing activity, but do not compete with ACE2 binding (Yuan et al., 2020a; Huo et al., 2020; Zhou et al., 2020; Lv et al., 2020; Wrobel et al., 2020). Although the left flank is largely separated from the neck and shoulders, two mAbs (38, 178) nevertheless compete and are situated closer to antibodies of the left shoulder, compared to more isolated antibodies (1, 22, 177) (Figure 2E). Some regions of the RBD are notable for the lack of antibody binding. The right and left flank clusters both interact with the neck and shoulder clusters, but this does not produce a complete “belt” of antibodies around the waist of the RBD. Antibodies are not seen against the N and C termini, either because of incomplete presentation on the RBD or occlusion by other parts of the spike.

### Mapping neutralization

In Figure 2F, we map neutralization to antibody position on the RBD. As expected, there is good correlation between overlap with the ACE2 footprint and neutralization. However, there were examples of non-neutralizing antibodies that were good ACE2 blockers, and it is not clear why these antibodies performed poorly. From the competition data, we can identify pairs of non-competing potently neutralizing mAbs and, if we relax the potency threshold, triplets (Table S3). Such combinations might prove useful in therapeutic cocktails (Baum et al., 2020; Dong et al., 2021).

There are undoubtedly mechanisms of neutralization beyond ACE2 blocking, for instance, 159 binds the NTD, remote from the ACE2 binding site (see below). Interestingly, antibodies co-locating with known neutralizing/protecting antibodies EY6A/H014 and S309 (Huo et al., 2020; Zhou et al., 2020; Lv et al., 2020) in the left and right flank clusters, respectively, did not show appreciable neutralization in our assays. We speculate that our assay might not be equally sensitive to all mechanisms of neutralization.

### Biophysical characterization of selected antibodies

We determined the kinetics of RBD attachment for 20 potent RBD binders (Table S2). K_D_ values for Fab fragments ranged from 0.7 to 7.6 nM and off-rates, potentially associated with therapeutic efficacy, were in the order of 1,000–10,000 s (Ylera et al., 2013). We also characterized expression levels, thermostability, monodispersity, and freeze-thaw robustness for 34 mAbs (Table S4). All were stable at elevated temperatures with a first observed Tm at 65°C–80°C (Walter et al., 2012) with more than 99% of the mass in a single species. A few appeared to have more complex unfolding pathways. Nearly all were resilient to 20 freeze-thaw cycles.

### Structural analysis of potent monoclonal antibodies, focusing on limited epitopes

**Figure 3:**
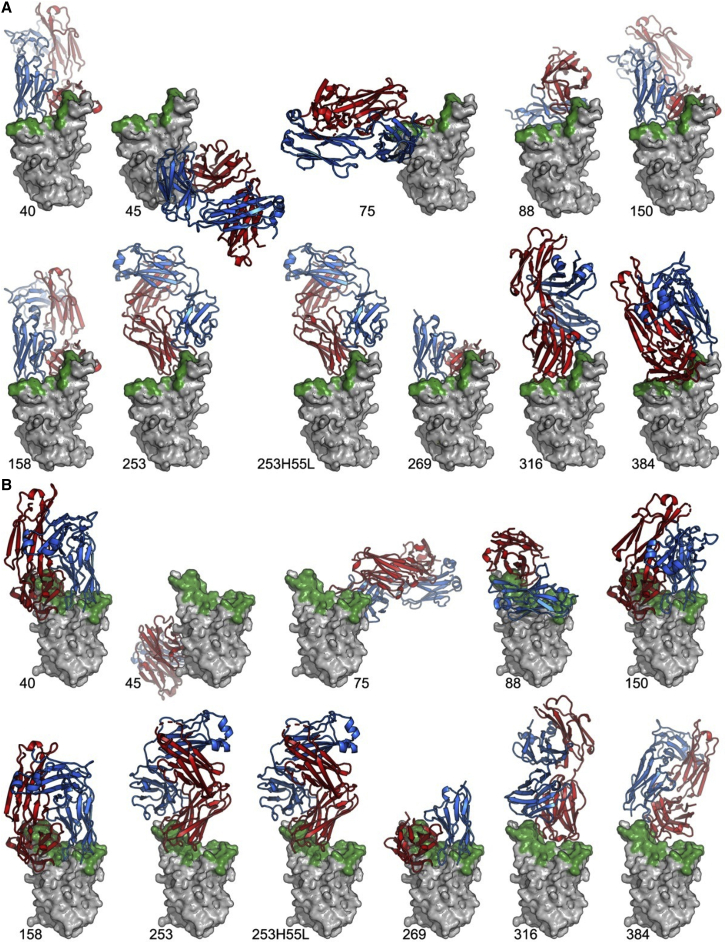
RBD complexes The Fab-RBD complexes reported in this paper as determined by a combination of X-ray crystallography with the exception of Fab 40 for which the Fab-RBD has been excised from a cryo-EM structure of Fab 40 bound to the S protein. (A) Shows the front view and (B) shows the back view with the RBD surface shown in gray and Fabs drawn as cartoons with the heavy chain in red and the light chain in blue. The ACE2 footprint on the RBD is colored in green. See also Figure S5.

**Figure 4:**
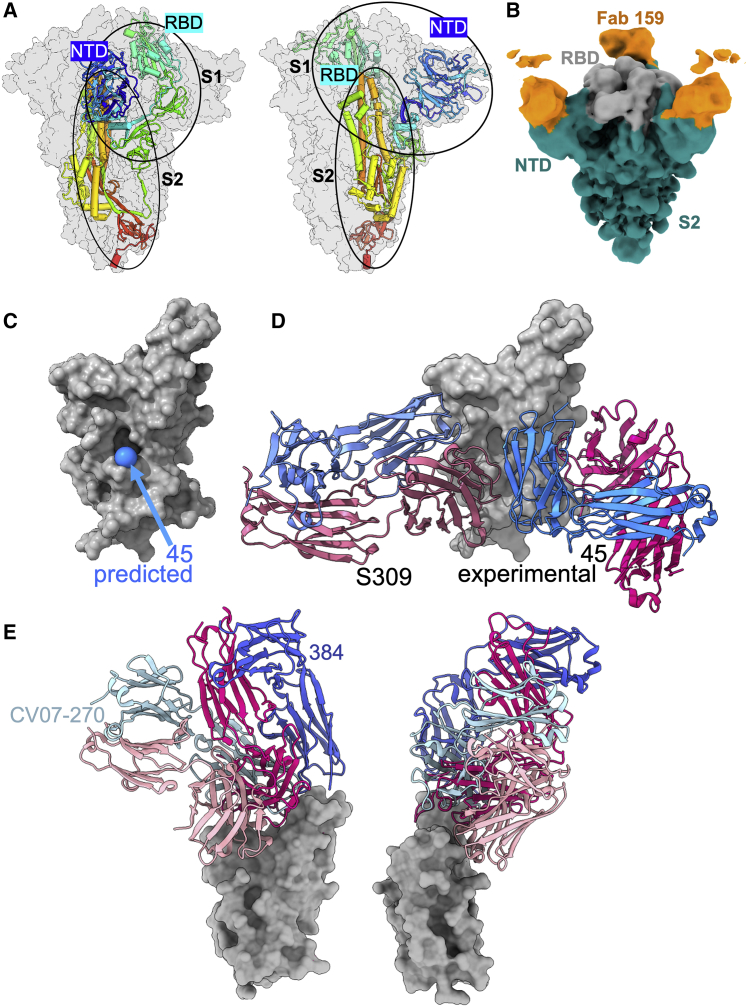
Spike morphology and Fab binding (A) Orthogonal views of the trimeric spike as a pale gray surface with one monomer depicted as a cartoon and rainbow colored from the N to the C terminus (blue to red). (B) Surface depiction of the electron potential map for the Spike-mAb 159 complex determined by cryo-EM to 4.1 Å resolution. The Spike is shown tilted forward and colored in teal apart from the RBDs (gray), and the fragment of mAb 159 that can be visualized is shown in orange. (C) Gray surface depiction of the RBD with a blue sphere denoting the location of Fab 45 as predicted using the mapping algorithm reported here. (D) Gray surface depiction of the RBD of the X-ray crystallographic structure of the observed RBD-Fab 45 complex. Fab 45 binds close to the predicted position but is slightly translated. The S309 Fab (the closest structure in the competition matrix on which the mapping algorithm was based) is shown superimposed. Both Fabs are depicted as a cartoon with the heavy chain in magenta and light chain in blue. (E) Orthogonal gray surface depictions of the RBD with Fab 384 bound and Fab CV07-270 superimposed onto the complex. These Fabs use the same heavy-chain V-gene but bind differently. They are drawn as cartoons with the heavy and light chains for Fab 384 in magenta and blue and those for CV07-270 in pale pink and light blue, respectively. See also Figures S4 and S5.

**Figure S4:**
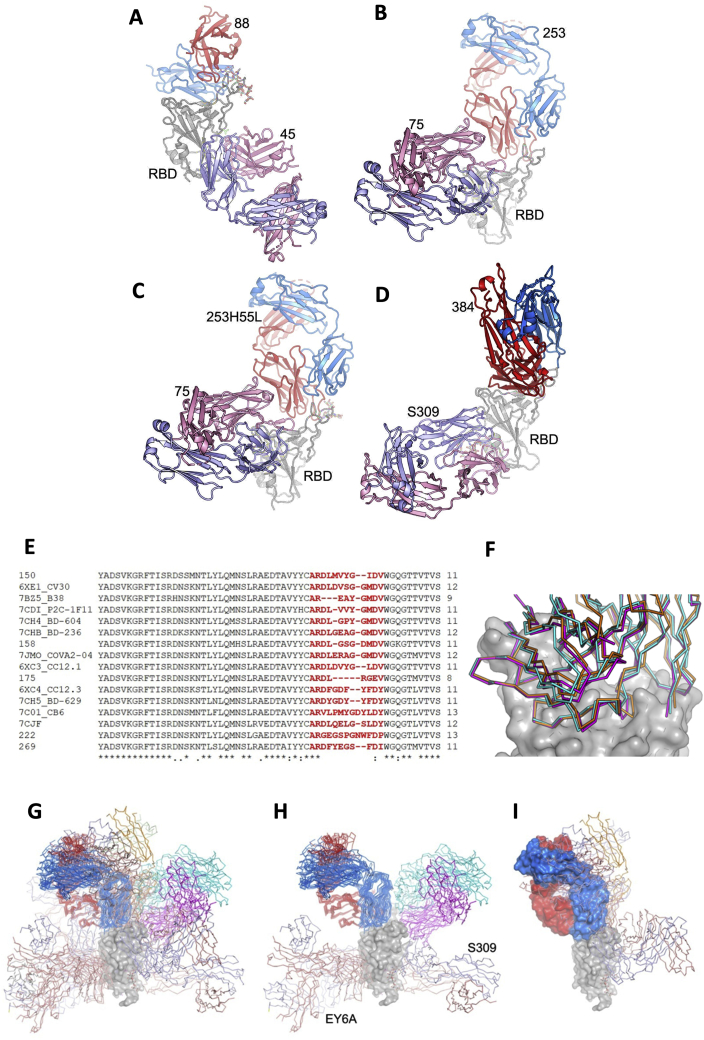
Crystal structures of the ternary complexes and overrepresentation of binding modes, related to Figures 4, 5, 6, and 7 (A) RBD-88-45, (B) RBD-253-75, (C) RBD-253H55L-75 and (D) RBD-384-S309 complexes. (E) Sequence alignment for HC CDR3s using public V-region 3-53, antibodies are represented by a number (from this study) or by PBD code and a name. (F) Comparison of binding modes of 150 (orange), 158 (cyan), 269 (magenta). (G) Superimposition of RBD-Fab complexes available in PDB (up to 21^st^ Oct. 2020). RBD is shown as gray surface, Febs as Cα traces with heavy chains in warm color and light chains in cool color. (H) The bound Fabs can be divided into four major clusters, neck (B38(7bZ5), CB6(7C01), CV30(6XE1), CC12.3(6XC4), CC12.1(6XC3), COV2-04(7JMO), BD629(7CHC), BD604(7CH4), BD236(7CHB)), left shoulder (p2b-2f6(7BWJ), BD368(7CHC), C07-270(6XKP)), left flank (EY6A(6ZCZ), CR3022(6YLA), S304(7JX3), COVA1-16(7JMW)) and right flank (S309 (7JX3)), according to their binding modes on RBD. (I) Outliers that include right shoulder binders (REGN10987 (6XDG), COVA2-39 (7JMP), CV07-250 (6XKQ), S2H14 (7JX3)). One Fab in the neck cluster is drawn as red and blue surface to show the relative position of the outliers.

**Figure S5:**
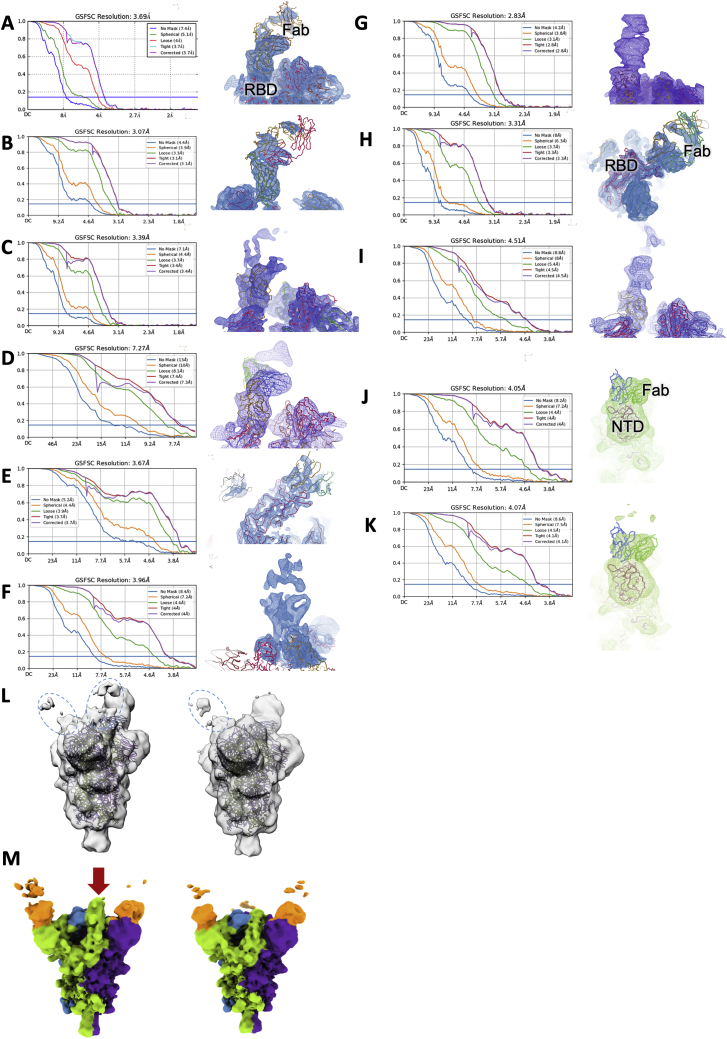
Cryo-EM data, related to Figures 3, 4, and 7 Resolution and map quality at the RBD-Fab/IgG interface. (A-K) [left] Gold-standard FSC curve (FSC = 0.143 marked) generated by cryoSPARC for fab (or IgG in the case of 159)-spike structures, [right] showing map quality at the antigen/antibody interface with 40, 88, 150, 158, 316, 384, 253H55L RBD up, 253H55L RBD down, 253H165L, 159 RBD down, 159 RBD up, respectively. Classification of Cryo-EM datasets shows Spike heterogeneity for 384 and 159. (L) Gaussian filtered reconstructed volume (transparent gray) with refined spike (from two clusters of 384 following local variability analysis using cryoSPARC). At very low contour levels, and with Gaussian filtering, we are able to see slight evidence of one (right), or two (left) additional bound fabs. (M) Reconstructed volume for 159 in the RBD up (left) and down (right) positions, colored by spike chain (blue, green, purple) and IgG (orange). The RBD in the up position is indicated by a red arrow.

Based primarily on the neutralization data (Table S2), we selected antibodies for structural analysis. Structures of 19 complexes, usually of either Fabs bound to isolated RBD (8, by crystallography) or of individual Fabs or mAbs bound to trimeric spike (11, by cryo-EM) were determined. Antibody 159 binds to the NTD, whereas all other antibodies bind the RBD (Figures 3, 4A, 4B, S4, and S5; Tables S5 and S6; STAR Methods). Many RBD-binders (40, 150, 158, and 269) bind to a tightly defined site in the neck cluster; 253, 316, and 384 bind more toward the front of the left shoulder; 88 binds toward the back of the left shoulder (although the footprints overlap); and mAb 75 binds at the right shoulder. The footprint of all of these antibodies overlaps with that of ACE2 (Figures 3, S4, and S5).

By selecting antibodies that are the most potent in the FRNT assay, we omitted a large number of high-affinity antibodies. This can be seen, for instance, in mAb 45, which had a K_D_ of 0.018 μg /mL. This mAb showed weak neutralization (IC_50_ 2 μg /mL) and was predicted as mapping to the right flank (Figure 4C). Structure determination of 45 in a ternary complex with potent neutralizer 88 and RBD revealed binding in the predicted position, a site not reported previously, adjacent to S309, an antibody with 79 ng mL^−1^ IC_50_ (Pinto et al., 2020; Piccoli et al., 2020) (Figures 3, 4C, and 4D), demonstrating the value of the predictive mapping in identifying novel epitopes.

### Potent antibody 384 binds in a previously unreported mode

**Figure 5:**
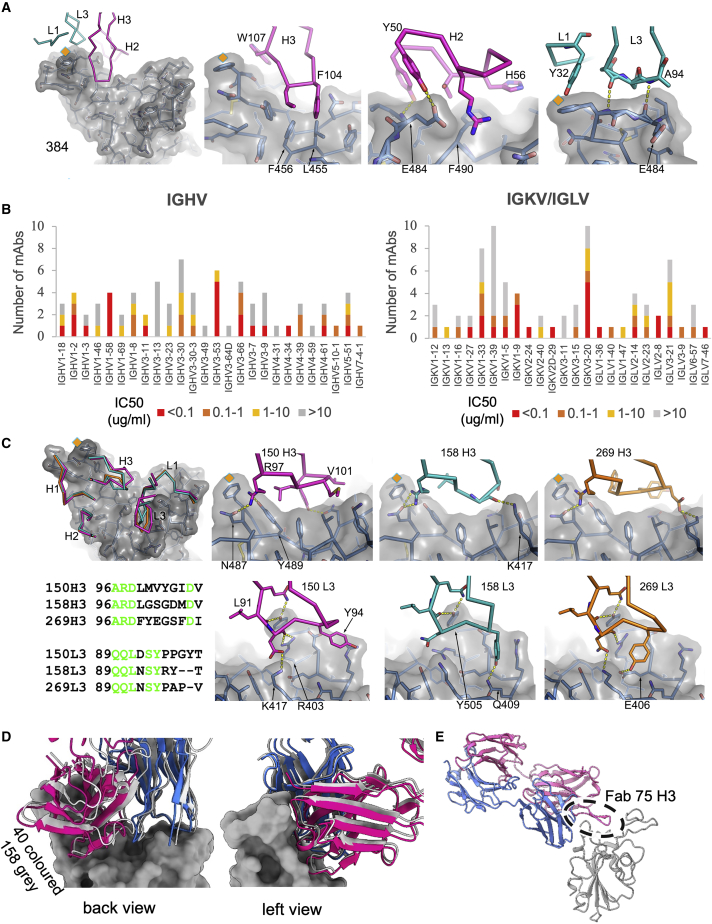
Determinants of binding, CDR length (A) Fab 384 interaction: left panel overview of the interacting CDRs from the heavy chain (magenta) and light chain (cyan) with the RBD (gray surface). The interactions of the H3, H2, and L1 and L3 loops are shown in the adjacent panels. (B) The distribution of IGHV, IGKV, and IGLV gene usage of anti-RBD antibodies. Antibodies are grouped and colored according to their neutralization IC_50_ values. (C) Left panel overview of the CDR interactions for Fabs 150 (magenta), 158 (cyan), and 269 (orange). Adjacent panels (top) show a close-up of the H3 loop interactions for each of these antibodies retaining the same color coding, and the bottom panel shows the interactions of the L3 loop and also the sequence alignment for the loops. (D) Back and side views of the complex of Fab 40 and RBD (gray surface) with the Fab drawn as a cartoon with the heavy chain in magenta and the light chain in blue. Fab 158 (gray cartoon) is superimposed. Note despite Fab 40 using the IGVH3-66 public V-gene, whereas 158 uses IGVH3-53 they bind almost identically. (E) Fab 75-RBD complex with the RBD drawn as a cartoon in magenta and the Fab similarly depicted with the heavy chain in orange and the light chain in gray. This antibody uses IGHV3-30 and is not a potent neutralizer. It can be seen that the only heavy-chain contact is via the extended H3 loop. See also Figures S3 and S4.

Antibody 384 is our most potently neutralizing mAb with an IC_50_ of 2 ng/mL. Its binding mode is unlike any other SARS-CoV-2 antibody reported to date. It approaches the binding site on the top of the neck and left shoulder from the front with a relatively small footprint of 630 Å^2^ (460 Å^2^ contributed by the heavy chain and 170 Å^2^ by the light chain). Although the orientation of 384 is similar to a group of previously reported Fabs (CV07-270, p2b-2f6, and bd629) (Kreye et al., 2020; Ju et al., 2020; Du et al., 2020), it is shifted 20 Å toward the left shoulder such that it does not contact the right chest (Figures 3 and 4E). Only CDRs H2 and H3 of the Fab 384 HC interact with the antigen (Figure 5A). It is unusual in that the 18-residue long H3 of Fab 384 binds across the top of the neck to reach the H3 binding site of the important IGVH3-53 group of Fabs (discussed below), making hydrophobic interactions from F104 and L105 at the tip to L455 and F456 of the RBD (Figure 5A). However, the main interactions that contribute to the binding affinity and orientation are with RBD residues 482–486 on top of the shoulder. W107 of H3 makes strong π-interactions with G485 and Y59 of H2, contacts V483, and makes bifurcated H-bonds to the carbonyl oxygen of G482 and amino nitrogen of E484. The latter also forms a salt-bridge with R52 and H-bonds to the side chains of T57 and Y59 (Figure 5A). E484–F486 also forms a two-stranded antiparallel β sheet with residues A92–A94 of L3 and makes stacking interactions from F486 to Y32 of L1.

### Repeated usage of heavy-chain V-regions demonstrates potent public responses

The potent neutralizers we have identified frequently use public HC V-regions (shared by most people, compared to private, patient-specific responses). Thus 5 potent mAbs use IGVH3-53 (bearing 3–10 non-silent mutations) (Figure 5B). IGVH3 antibodies have been observed before (e.g., B38, CB6, and CC12.3) (Wu et al., 2020a, 2020b; Shi et al., 2020; Yuan et al., 2020b; Hurlburt et al., 2020; Du et al., 2020; Clark et al., 2020). Our competition data showed that these all bind at a similar site. We determined structures for three members of the group, 150, 158, and 269 (the others are 175 and 222) and found that they bind almost identically at the back of the neck with similar footprints of ~800 Å^2^ (Figures 3 and S4B). The flat binding site of the RBD and the approach angle of the Fabs limit their H3 length (11 residues) and the number of contacts H3 makes with the RBD (Figure 5C), which is compensated for by the interactions from H1, H2, and all CDRs of the light chain. Thus for 158, H3 makes four direct contacts (≤4 Å) and two hydrogen bonds to the RBD, whereas H1 and H2 together make 11 contacts and 6 hydrogen bonds, and the three LC CDRs contribute 6 contacts and 5 hydrogen bonds (Figure 5C). We note strong LC interactions with residue N501 of the RBD, which is mutated in recent variants (B.1.1.7, B.1.351, P.1). The H3 length matches that reported as optimal for this V-region (Yuan et al., 2020b), and the H3 sequence of mAb 150 is strongly similar to that of CC1.12 (Yuan et al., 2020b) (Figure S4A). Thus, H1 and H2 determine the mode of engagement, as seen in previous studies of antibodies with this V-region (Figure S4C) (Yuan et al., 2020b).

A second V-region that repeatedly confers potent (IC_50_ < 0.1 μg/mL) neutralization is IGVH1-58 (mAbs: 55, 165, 253, and 318). These have even fewer non-silent mutations (2–5) and longer HC CDR3s (12–16 residues). Three antibodies (55, 165, and 253) harbor a disulfide bond in their CDR3s, compete strongly with each other for binding, and map to the neck epitope but do not compete with mAb 318. In mAb 253, the disulfide brackets a glycosylation sequon (see below). The crystal structure of a complex including Fab 253 confirmed that it binds within the dominant neck epitope (Figure 3). In contrast, competition mapping indicates that Fab 318 binds at the right shoulder epitope (Figure 2E). It appears that for this V-region, the CDR3 is more critical to recognition and can switch binding to different epitopes on the same antigen but nevertheless can bind strongly with near germline V-region sequences.

The final V-region with at least 2 potent neutralizers is IGHV3-66, which was found a total of 5 times with 2 potent neutralizers (282 and 40). These two (with rather few mutations from germline and CDR3 lengths 12 and 13, respectively) compete strongly. Once again, we determined a complex structure for one (Fab 40) and demonstrated that, as expected from the competition data, this antibody binds squarely in the dominant neck epitope, almost indistinguishable from those using IGHV3-53 (Figure 5D). One IGHV3-66 mAb (398) has a much longer H3, 21 residues, and is predicted to bind on the edge of the neck epitope (Figure 2E).

IGHV3.11 is found in the most potent neutralizer, 384 but is also used by CV07-270 (Kreye et al., 2020). CV07-270 is swung forward and sideways (compared to 384) (Figure 4E) so that it does not compete with ACE2 binding, suggesting that the potency of 384 derives from the extended H3 interaction that reaches across the ACE2 binding site.

Although IGHV3-30 is found in 11 RBD binders, none are potent neutralizers. H3 lengths for IGHV3-30 RBD binders vary from 12 to 20 residues, suggesting they bind at different sites, as confirmed by the structures of two representatives, 75 (in a ternary complex with 253) and 45 (in a ternary complex with 88) (Table S5). 75 binds on the right shoulder and overlaps the ACE2 binding site (Figure 3), however, the only HC-RBD contact is via the extended 20 residue H3, whereas the bulk of the interaction is with the LC, outside of the ACE2 footprint, and ACE2 binding could likely displace the extended H3 loop (Figure 5E). 45, with an H3 length of 14 residues, binds differently, well away from the ACE2 binding site on the left flank and so would not be expected to neutralize (Figure 3). Thus, for IGHV3-30 antibodies, the mode of binding is modulated by H3 and not focused on a region overlapping the ACE2 site.

In summary, the major public V-regions used by potent antibodies generally target the neck epitope, usually with a common mode of binding dictated by the V-region (although they can occasionally switch epitopes), but this is not true for weaker neutralizers. This likely explains the overwhelming representation of a common mode of binding at the neck epitope in the structures determined to date (Figure S4C).

### Light-chain mixing can increase neutralization titer

**Figure 6:**
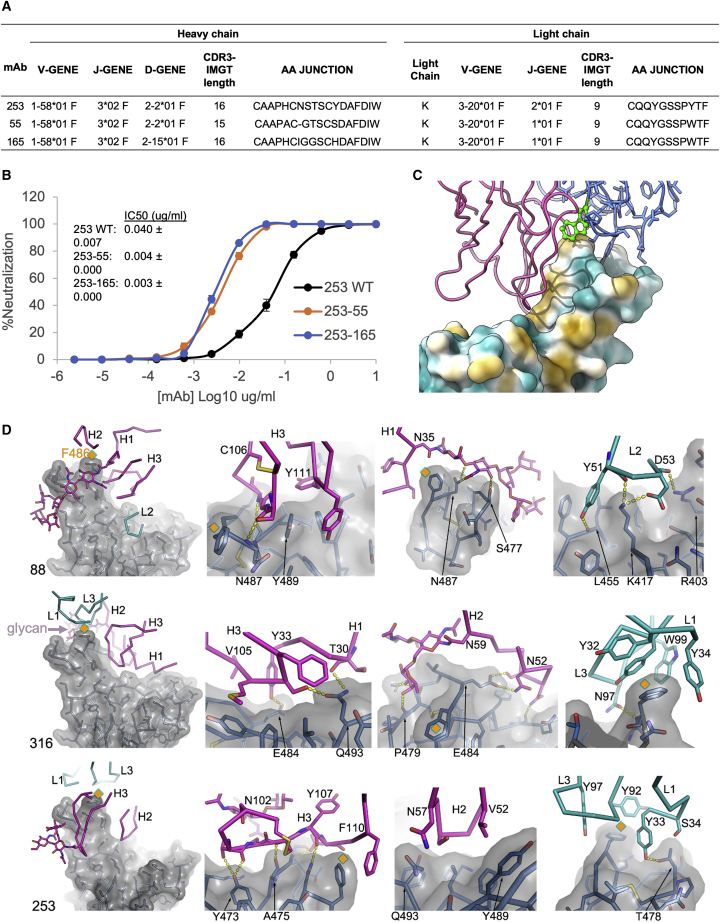
Determinants of binding, light-chain swapping, and glycosylation (A) Table of sequences of mAbs 253, 55, and 165. (B) Neutralization activity of authentic SARS-CoV-2 by the original mAb253, chimeric mAb253H55L, and chimeric 253H165L (presented as IC_50_ values). Immunoglobulin heavy- and light-chain gene alleles are presented in the table. Data are from 3 independent experiments, each with duplicate wells and the data are shown as mean ± SEM. (C) The chimeric Fab 253H55L (mAb 253 [IGVH1-58] heavy chain combined with the light chain of mAb 55 [IGVK3-20]) in complex with the RBD here shown as a hydrophobic surface. The Fab is drawn as a ribbon with the heavy chain in magenta and the light chain in blue. This 10-fold increase in neutralization titer of this Fab compared to 253 appears to come from the single substitution of a tryptophan for a tyrosine making a stabilizing hydrophobic interaction. (D) CDRs with sugar bound in the RBD complexes with Fabs 88 (top panel) sugar bound to N35 in the H1 loop, 316 (middle panel) sugar bound to N59 in the H2 loop, and 253 (bottom panel) sugar bound to N102 in the H3 loop. Note that Phe 486 is marked by a diamond to enable the various orientations to be related. See also Figures S4 and S6.

For the three potent anti-RBD antibody clusters where >2 members shared the same IGVH (IGHV3-53, IGHV1-58, and IGHV3-66), we performed a mixing experiment, where each IGVH was matched with all the IGVL within that cluster (Figure 6A). Chimeric antibodies were expressed, and neutralizations were performed and compared with the original mAb clone. Unexpectedly, we found a 10-fold increase in neutralization titers when the heavy chain of mAb 253 (IGVH1-58, IGVK3-20) was combined with the light chain of either mAb 55 or 165, which have the same V-gene pairing (IGVH1-58, IGVK3-20) but a different J gene, having IGKJ1 in contrast of IGKJ2 in mAb 253 (Figure 6B). Remarkably the sole difference in contact residues is a Trp for Tyr substitution in mAbs 55 and 165 (Figure 6C). Structural analyses of Fab-complexes with RBD reveals the large hydrophobic tryptophan side chain stabilizing a hydrophobic region of the antibody and nestled against the key hydrophobic region (E484–F486) of the RBD used by many potent neutralizers, whereas the smaller tyrosine side chain makes fewer contacts.

### The role of N-linked glycan in antibody interaction

**Figure S6:**
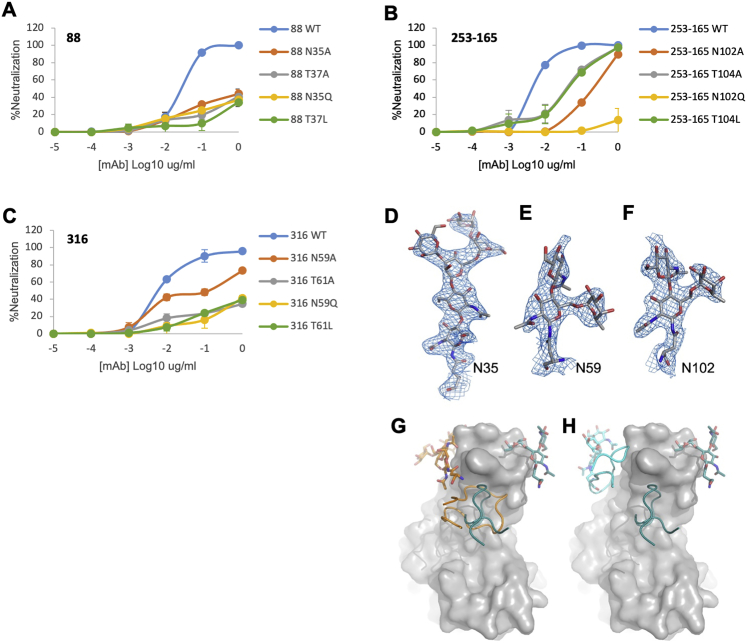
Importance of antibody glycosylation, related to Figure 6 (A-C) Effect of mutation of the Asn residue glycosylated in the heavy chains of antibodies 88, 253 and 316 respectively. (D-F) |2Fo-Fc| electron density maps contoured at 1.2 σ showing the glycans at glycosylation sites at N35 of 88 (D), N59 of 316 (E) and N102 of 253 (F). (G) Relative binding position and orientation of CDR-H3 and glycans between 316 (green) and 88 (orange), and (H) between 316 and 253 (cyan). RBD is shown as a gray surface.

Although 15%–25% of IgGs bear N-linked glycans in their variable regions, sometimes with impact on antigen binding, this finding is relatively poorly studied at the molecular level (Wright et al., 1991; van de Bovenkamp et al., 2016). Of 80 RBD-binding antibodies described here, 14 (17.5%) contain glycosylation sequons arising from somatic mutations in their variable region. For 8 mAbs (1, 88, 132, 253, 263, 316, 337, and 382) the sequons are in the HC, and for 5 mAbs, they lie in a CDR. Several of the HC mutations, but none of the LC mutations, are in potently inhibitory antibodies (neutralization IC_50_ <0.1 μg/mL). Two of these (88 and 316) could be de-glycosylated without denaturation, and BLI analysis showed that this had negligible effect on RBD/Fab affinities (K_D_ = 0.8/1.2 nM and 1.0/2.0 nM, de-glycosylated/glycosylated, respectively, for 88 and 316), although the on-rate was a little faster in the absence of sugar (e.g., 3.8 × 10^5^ 1/Ms [megasecond] compared to 1.4 × 10^5^ 1/Ms for mAb 88). However, mutations that eliminate glycosylation had a deleterious effect on neutralization for these two and for the 253H165L chimera (Figure S6). Structures were therefore determined for mAbs 88, 316, and 253 in complex with RBD and with spike (Figures 3, 6D, and S6; Tables S5 and S6).

Antibodies 88 and 316 contain glycosylation sites in H1 (N35) and H2 (N59), respectively. The crystal structure of the RBD-316 Fab complex at 2.3 Å resolution shows well-defined density for 3 glycans including an α1,6-linked fucose (Figures 6D and S6E). The structure of Fab 88 was determined in a ternary complex with 45 and RBD to 2.53 Å resolution (the ChCl domains of 88 were disordered, but the VhVl domains had well-defined density). Antibody 88 binds to the back of the neck whereas 316 binds to the top of the neck, orientated radically differently, however, the H3s of the two Fabs overlap well (Figures 6D and S6). The glycans of Fab 88 surround the back of the left shoulder like a necklace and those of Fab 316 sit on the top of the same shoulder. Fab 88 has a footprint of 1,110 Å^2^ (390 Å^2^, 420 Å^2^, and 300 Å^2^ from HC, LC, and glycans, respectively), whereas Fab 316 has a footprint of 950 Å^2^ (610 Å^2^, 150 Å^2^, and 190 Å^2^ from HC, LC, and glycans, respectively). As we describe above for mAb 384, residues E484–F486 of the RBD make extensive interactions in these antibodies with residues from the 3 CDRs of the HC and L1 and L3 of the LC, thus for 316 the side chain of E484 H-bonds to N52 and S55 of H2 and Y33 of H1, G485 contacts W50 of H2, and F486 makes strong ring stacking interactions with Y93 and W99 of L3 and Y34 of L1. This suggests E484–F486 constitutes a hot-spot of the epitope. These residues are accessible from a variety of different angles of attack, thus Fabs 384, 316, and 88 all interact with this region despite their markedly different poses on the RBD. In contrast, the H3 of 253 overlaps with the glycans of mAb 88, and the glycan of mAb 253 makes no direct interactions with the RBD (Figure 6D).

In all cases, the sugar is presented close to the top of the left shoulder and, in 2 out of 3 cases, interacts directly but rather weakly with the antigen. The high frequency of sequon generation despite the rather few somatic mutations is intriguing and suggests positive selection.

### Binding in the context of the trimeric spike

**Figure 7:**
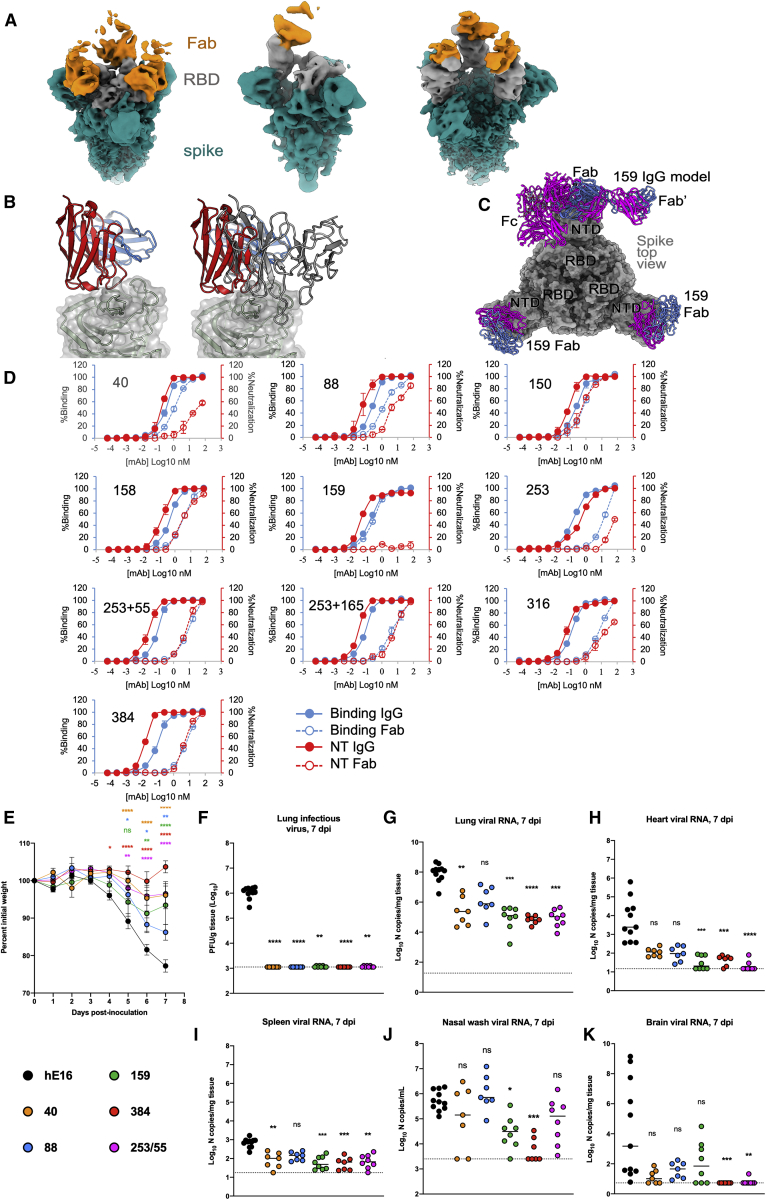
Determinants of binding, valency of interaction, and *in vivo* studies (A) Cryo-EM Spike-Fab complexes showing different RBD conformations. The density for the Spike is shown in teal, the RBD in gray, and Fab in orange. Left: “all RBDs down” conformation with Fab 316 bound. Middle: “one RBD up” conformation with one Fab 158 bound. Right: “all RBDs up” conformation with 3 Fab 88s bound. (B) Left: potently neutralizing Fab 159 (cartoon representation with red heavy chain and blue light chain) in complex with the NTD (gray transparent surface). Right: 159 is depicted with another NTD binding Fab (4A8) superimposed as a gray ribbon, the binding sites are separated by ~15 Å. (C) Fab 159 (magenta, HC; blue, LC) is drawn as a cartoon in its binding location on top of the NTD of the Spike that is drawn as a gray surface and viewed from the top (a full IgG is modeled onto one monomer showing that it cannot reach across to bind bivalently). (D) ELISA binding (blue) and FRNT neutralization (red) curves of ten full-length antibodies (solid lines) and corresponding Fab molecule (dash lines) against SARS-CoV-2. Data are from 2 independent experiments (mean ± SEM). (E–K) Seven- to 8-week-old male and female K18-hACE2 transgenic mice were inoculated by an intranasal route with 10^3^ PFU of SARS-CoV-2. At 1 dpi, mice were given a single 250 μg (10 mg/kg) dose of the indicated mAb by intraperitoneal injection. (E) Weight change (mean ± SEM; n = 5–10, two independent experiments: two-way ANOVA with Sidak’s post-test: ns, not significant, ^∗^p < 0.05, ^∗∗^p < 0.01, ^∗∗∗∗^p < 0.0001; comparison to the isotype control mAb treated group). (F–K) At 7 dpi tissues were harvested and viral burden was determined in the lung (F and G), heart (H), spleen (I), nasal washes (J), and brain (K) by plaque (F) or qRT-PCR (G–K) assay (n = 7–11 mice per group; Kruskal-Wallis test with Dunn’s post-test: ns, not significant, ^∗^p < 0.05, ^∗∗^p < 0.01, ^∗∗∗^p < 0.001, ^∗∗∗∗^p < 0.0001). Dotted lines indicate the limit of detection. See also Figures S4, S5, and S7.

On isolated stabilized spikes the RBD is found in two orientations; “up” and “down” (Yuan et al., 2017; Roy et al., 2020). Both of these form an ensemble of conformations, up conformations vary by up to 20° (Zhou et al., 2020) and down can include a tighter packed “locked” conformation (Ke et al., 2020; Toelzer et al., 2020; Carrique et al., 2020; Xiong et al., 2020). The structures we see by cryo-EM have the RBD in either the classic up or down conformation (see Figure 7A), although antibody binding sometimes introduces small perturbations in the RBD orientation. The most common configuration observed for the spike construct we have used is 1 RBD-up and 2-down. ACE2 can only attach to the up conformation, which is assumed to be less stable, favoring conversion to the post-fusion state. In our structures, we see Fabs 40, 150, 158, and the chimeras 253H55L and 253H165L binding to the spike in this one-up configuration. 253H55L also binds to the all-down configuration (1 Fab/trimer), as does Fab 316 (3 Fabs/trimer) and Fab 384 (1 Fab/trimer). In contrast, Fab 88 binds (3 Fabs/trimer) in the all-up configuration (Figure 7A; Table S6).

Although Fab 384, despite its high potency, predominantly binds only one RBD per trimer, analysis of different particle classes revealed some weak density decorating the other RBDs, also in the down position, whereas a subtle movement can be seen between the RBDs of different classes (Figure S5L). This could be attributed to a more favorable RBD conformation that can only be sustained by one RBD at a time.

To visualize the binding of the highly potent mAb 159, it was necessary to incubate spike with 159 IgG (the Fab alone showed no binding). This revealed all three NTDs of the spike decorated by 159 with RBDs in either one-up or all-down configurations (Figure S5M). The 159 binding site is ~15 Å from that of a previously reported NTD binder, 4A8 (Chi et al., 2020), in which the CDR-H3 binds on the side of the NTD between the 144–153 and 246–258 loops (Figure 7B). The CDR-H3 of 159 is 11 residues shorter than that of 4A8 (Chi et al., 2020) and binds on the top center of the NTD interacting with residues 144–147, 155–158, 250–253, and the N terminus of NTD. All 3 CDRs of the heavy chain contribute to a foot print of 515 Å^2^ on the NTD, whereas the light chain has little contact with the NTD (35 Å^2^), similar to 4A8 (Chi et al., 2020) (Figures 7B and 7C).

### Valency of interaction

We measured binding of full-length mAbs and Fab fragments to intact SARS-CoV-2 by ELISA and compared these with neutralization curves for antibodies for which we have structural information (Figure 7D; Table S7). For the anti-NTD, mAb 159 binding of full-length and Fab to virions were nearly identical, in line with NTDs on a trimer being too far apart to allow bivalent engagement (118 Å) (Figure 7C) and suggesting that mAb 159 cannot span adjacent spike trimers at the virion surface. Interestingly, although IgG 159 is a potent neutralizer, Fab 159 has no neutralizing activity, suggesting that the Fc portion is crucial for activity, although the mechanism is not immediately apparent and does not involve blocking ACE2 interaction.

Loss in binding and neutralization with Fabs compared to IgG is quite modest for mAb 88, which attaches in the all-up conformation (Figures 7D and S6), but much more marked for mAbs that bind the all-down form of the spike (253, 316, and 384). Thus, mAb-384 showed 79-fold less virus binding and a 486-fold loss of neutralization activity when reduced to Fab, suggesting that both Fab arms are used when antibody interacts with virions and also highlights the exceptional K_D_ of Fab-159, 2.5- to 81-fold better than the other Fabs depicted in Figure 7D and Table S7. Finally, we have used the following formula to estimate the relationship between antibody binding and neutralization: percent occupancy = BMax^∗^ [Ab]/(Kd + [Ab]), where the BMax is percent maximal binding, [Ab] is the concentration of Ab required to reach 50% FRNT, and Kd is the concentration of Ab required to reach half-maximal binding. mAb-384 can achieve NT50 with an estimated average occupancy of 12% of the maximum available antibody binding sites on each virion, perhaps in part due to the avidity conferred by bivalent attachment (Table S7). Bivalent attachment to the down conformation may also lock all three RBDs, preventing attachment to ACE2. Some of the variation in the effects seen in Figure 7D and Table S7 probably arises from the interplay between the angle and position of attack of the antibody arm to the RBD and the constraints on flexibility in the system.

### *In vivo* efficacy

**Figure S7:**
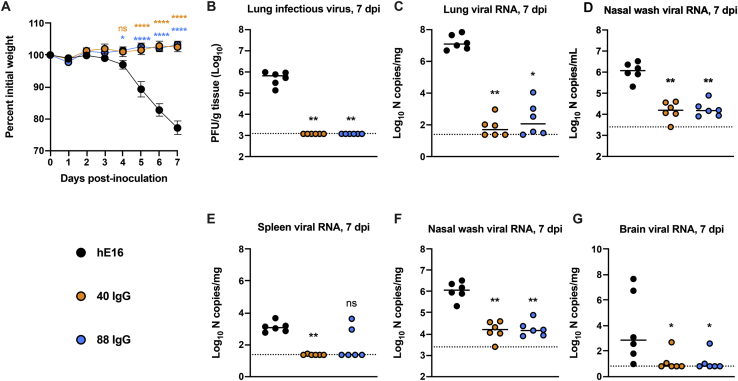
Prophylaxis with mAbs 40 and 88 protects against weight loss and decreases viral burden, related to Figure 7 **(**A-G) Seven to eight-week-old male and female K18-hACE2 transgenic mice were given a single 250 μ dose of the indicated mAbs by intraperitoneal injection. One day later mice were inoculated by intranasal route with 10^3^ PFU of SARS-CoV-2. (A) Weight change (mean ± s.e.m; n = 6, two independent experiments: two-way ANOVA with Sidak’s post test: ns, not significant, ^∗^p < 0.05, ^∗∗∗∗^p < 0.0001; comparison is to the isotype control mAb treated group). B-G. At 7dpi tissues were harvested and viral burden was determined in the lung (B-C), heart (D), spleen (E), nasal washes (F) and brain (G) by plaque assay (B) or RT-qPCR (C-G) assay (n = 6 mice per group. Kruskal-Wallis test with Dunn’s post-test: ns, not significant, ^∗^p < 0.05, ^∗∗^p < 0.01, ^∗∗∗^p < 0.001). Dotted lines indicate the limit of detection.

We determined the efficacy of our most promising neutralizing human mAbs *in vivo*. We utilized the K18-hACE2 transgenic mouse model of SARS-CoV-2 pathogenesis, wherein human ACE2 expression is driven by an epithelial cell-specific, cytokeratin-18 gene promoter (McCray et al., 2007; Winkler et al., 2020). In this model, SARS-CoV-2 infected animals develop severe pulmonary disease and high levels of viral infection in the lung that is accompanied by immune cell infiltration and tissue damage (Winkler et al., 2020). Initially, a single 250 μg (10 mg/kg) dose of mAbs 40 and 88 were administered as prophylaxis by intraperitoneal injection 1 day prior (D−1) to intranasal (i.n.) challenge with 10^3^ plaque-forming unit (PFU) of SARS-CoV-2. Passive transfer of mAb 40 or 88, but not an isotype control mAb (hE16), prevented SARS-CoV-2-induced weight loss (Figure S7A). In the lung homogenates of antibody 40- and 88-treated animals, no infectious virus was detected at 7 dpi, whereas substantial amounts were present in animals treated with the isotype control mAb (Figure S7B). Consistent with these results, viral RNA levels were reduced by ~10,000- to 100,000-fold compared to isotype control mAb-treated animals (Figure S7C). In peripheral organs, including the heart, spleen, or brain, viral RNA levels were reduced or undetectable in mAb 40- or 88-treated animals (Figures S7D–S7G). Moreover, levels of viral RNA at 7 dpi were markedly lower in the nasal washes of animals treated with mAbs 40 and 88 compared to the isotype control.

To further evaluate the *in vivo* potency of our mAbs, we assessed the therapeutic activity of a larger panel at 1 dpi (D+1) with 10^3^ PFU of SARS-CoV-2. Although varying degrees of protection were observed for individual mAbs, weight loss was significantly reduced in all animals treated with anti-SARS-CoV-2 mAbs at 6 and 7 dpi compared to the isotype control (Figure 7E). Whereas the lungs of isotype control mAb-treated animals had infectious virus levels of ~10^6^ PFU/g of tissue, we barely detected infectious virus in animals treated with the mAbs 40, 88, 159, 384, or 253H55L (Figure 7F). Lung viral RNA levels at 7 dpi also were reduced in animals treated with mAbs 40, 159, 384, and 253H55L, although statistical significance was not achieved with mAb 88 despite mean reductions of ~100-fold (Figure 7G). At sites of disseminated infection, notably the heart, spleen, and brain, all anti-SARS-CoV-2 mAbs showed protective activity, although mAbs 384 and 253H55L conferred the greatest reductions in viral RNA levels (Figures 7H–7K). In nasal washes, mAbs 159 and 384 showed the best ability to reduce viral RNA levels (Figure 7J). Collectively, these data demonstrate several mAbs in our panel can reduce infection in the upper airway, lower airway, and at distant sites when administered after infection.

## Discussion

There is now a substantial database of antibody/antigen complexes for the SARS-CoV-2 spike (84 PDB depositions as of 12 December 2020, including nanobody structures). The number of unique structures is smaller than this, and the focus on potently neutralizing public V-regions means that many have near identical binding modes (Figure S4). Here, we report, in contrast, a comprehensive analysis of anti-SARS-CoV-2 human mAbs. We measured the neutralization ability of a set of 377 mAbs from a substantial cohort of COVID-19 patients and identified that 80 of these bind the RBD. We have determined the binding sites for all 80 of these, using a combination of structural methods and a novel computational algorithm based on bio-layer interferometry competition measurements. This defines five binding clusters or epitopes. By analogy with a human torso, four of these clusters form a continuous swathe running from the left shoulder to the neck, right shoulder, and down the right flank of the torso whereas the fifth cluster forms a more discrete site toward the left flank. These sites are widely distributed over the surface, however, all but one of the 20 most potent (IC_50_ <0.1 μg/mL) neutralizing mAbs block receptor attachment to the neck. The single exception, mAb 159, binds the NTD and the mechanism of neutralization is unclear.

The large body of structural results allowed us to dissect the high-resolution details of binding of the major classes of potent neutralizers that bind the RBD. Highly potent ACE2 blocking mAbs map to two sites in the region of the neck and left shoulder, residues E484–F486 bridge the epitopes and are accessible to Fabs binding from a variety of different angles of attack. It is notable that mutation F486L has been identified as a recurrent mutation associated with host-adaptation in mink (van Dorp et al., 2020), and likewise, mutation E484K is found in the recently identified B.1.351 and P.1 lineages. We would expect these changes to impact on the binding of many of our most potent mAbs, including 384. A characterization of the polyclonal antibody response would give insight into the potential for vaccine escape.

There is a close association between potent neutralizers and public V-genes suggesting that vaccination responses should be strong (Barnes et al., 2020, Yuan et al., 2020b). Three public V-region genes are represented at least twice in our set, (1) IGHV3-53: mAbs 150, 158, 175, 222, and 269; (2) IGHV1-58: 55, 165, 253, and 318; and (3) IGHV3-66: 282 and 40. The potent binders focus around the neck cluster, often with binding pose determined by the H1 and H2 loops. By switching light chains within these sets, we found that one (253) could improve functionally by an order of magnitude by using an alternate light chain to achieve better hydrophobic interactions with the key bridging region we identify, E484–F486. The most highly potent mAb, 384, adopts a unique pose, with a footprint extending from the left shoulder epitope across to the neck epitope via an extended H3.

Despite the most potently neutralizing mAbs being close to germline, somatic mutations introduce N-linked glycosylation sites into the variable region of 17.5% of the potent neutralizers. These can contribute to the interaction with the RBD, and although they appear to have relatively little effect on affinity, they significantly enhance neutralization. The enhanced neutralization we observe (along with other favorable properties, e.g., solubility, stability, and mitigation of auto-antigen responses) warrant investigation of production methods to allow variable region glycosylated Fabs to routinely meet regulatory standards (Zhang et al., 2016).

We find that there is a correlation between Fab versus IgG binding/neutralization and the mode of attachment to the prefusion spike as seen by cryo-EM. Those antibodies that bind the spike in the down conformation appear to show a marked avidity boost to binding and neutralization when Fab and full-length IgG1 are compared (e.g., 316 and 384), suggesting that there is a relationship between the mode of attachment and neutralization that is still not fully understood, as also seen from the potent neutralization reported for antibodies that bind at the left and right flank (S309 and EY6A/H014) (Pinto et al., 2020; Zhou et al., 2020; Lv et al., 2020) epitopes that do not report strong neutralization in the assay we use in this report.

Finally, we demonstrate that the most potent antibodies we have identified can protect in an animal model, when administered prophylactically or therapeutically. The competition mapping method we have devised suggests a series of combinations of neutralizing antibodies with non-overlapping epitopes that could create an immunotherapy with greater protection and resistance against mutation than a single monoclonal antibody.

### Limitations of the study

The mechanisms of neutralization by antibodies that bind to the NTD are not yet established and will be the subject of further study. The correlates of protection from SARS-CoV-2 infection have not yet been established, and the role of T cells needs further study. It is also the case that *in vitro* neutralization assays do not capture the contributions of Fcγ receptor interactions and complement activation that likely contribute to protection *in vivo*. The mapping methodology could be improved, for instance, by covalently anchoring the antigen, by more complete sampling, or by better modeling of the antibody shape. It could also be made more routine by higher density testing (e.g., 384-well plates). However, the approach could be applied equally to other data (e.g., surface plasmon resonance or ELISA) to provide a general way of rapidly determining locations from highly redundant competition experiments.

## STAR★Methods

### Key resources table

**Table:** 

REAGENT or RESOURCE	SOURCE	IDENTIFIER
**Antibodies**		

Fab	This paper	N/A
IgG	This paper	N/A
Human anti-NP (mAb 206)	This paper	N/A
Mouse anti-SARS-CoV-2 spike (mAb31 with murine Fc)	This paper	N/A
StrepMAB-Classic	iba	Cat#2-1507-001; RRID:AB_513133
StrepMAB Classic DY549	iba	Cat#2-1566-050
Anti-Human CD3-FITC	BD	Cat#555332; RRID:AB_395739
Anti-Human CD14-FITC	BD	Cat#555397; RRID:AB_395798
Anti-Human CD56-FITC	BD	Cat#562794; RRID:AB_2737799
Anti-Human CD16-FITC	BD	Cat#555406; RRID:AB_395806
Anti-Human IgM-FITC	BD	Cat#555782; RRID:AB_396117
Anti-Human CD19-BUV395	BD	Cat#563549; RRID:AB_2738272
Anti-Human IgG-BV786	BD	Cat#564230; RRID:AB_2738684
Anti-Human IgM-APC	BD	Cat#551062; RRID:AB_398487
Anti-Human IgA-FITC	Dako	Cat#F0188
Anti-Human IgD-FITC	Dako	Cat#F0189
Anti-Human IgG (Fab-specific)-ALP	Sigma	Cat#A8542; RRID:AB_258397
Anti-Human IgG (Fc-specific)-ALP	Sigma	Cat#A9544; RRID:AB_258459
Anti-Human IgG (Fc specific)-Peroxidase	Sigma	Cat#A0170; RRID:AB_257868
Anti-human IgG Fc specific-FITC	Sigma	Cat#F9512; RRID:AB_259808
anti-mouse IgG Fc-AP	Invitrogen	Cat#A16093; RRID:AB_2534767
Tetra-His antibody	QIAGEN	Cat#34670; RRID:AB_2571551
FD7C antibody	Huang et al., 2020	N/A

**Bacterial and virus strains**		

SARS-CoV-2 (Australia/VIC01/2020)	Caly et al., 2020	N/A
2019 n-CoV/USA_WA1/2020	US CDC	N/A
DH5α bacteria	*In Vitro*gen	Cat#18263012
Rosetta-gami 2(DE3)pLysS bacteria	Sigma-Aldrich	Cat#71352

**Biological samples**		

PBMCs from SARS-CoV-2 patients	John Radcliffe Hospital in Oxford UK	N/A

**Chemicals, peptides, and recombinant proteins**		

His-tagged SARS-CoV-2 RBD	This paper	N/A
Biotinylated SARS-CoV-2 RBD	This paper	N/A
His-tagged human ACE2	This paper	N/A
Human ACE2-hIgG1Fc	This paper	N/A
SARS-CoV-1 Spike	This paper	N/A
MERS-Cov Spike	This paper	N/A
OC63-CoV Spike	This paper	N/A
HKU1-CoV Spike	This paper	N/A
229E-CoV Spike	This paper	N/A
NL63-CoV spike	This paper	N/A
SARS-CoV-2 Spike	This paper	N/a
His-tagged SARS-CoV-2 NP	This paper	N/A
SARS-CoV-2 spike S1	Sino Biological	Cat#40591-V08H
SARS-CoV-2 spike S2	Sino Biological	Cat#40590-V08B
Phosphate buffered saline tablets	Sigma-Aldrich	Cat#P4417
Biotinylation kit	Avidity	Cat#BirA500
Sensor Chip Protein A	Cytiva	Cat#29127555
Dulbecco’s Modified Eagle Medium, high glucose	Sigma-Aldrich	Cat#D5796
Dulbecco’s Modified Eagle Medium, low glucose	Sigma-Aldrich	Cat#D6046
FreeStyle 293 Expression Medium	GIBCO	Cat#12338018
L-Glutamine–Penicillin–Streptomycin solution	Sigma-Aldrich	Cat#G1146
Fetal Bovine Serum	GIBCO	Cat#12676029
Polyethylenimine, branched	Sigma-Aldrich	Cat#408727
Recombinant RBD-mFc	Sino Biological	Cat#40592-V05H
Streptavidin-APC	Biolegend	Cat#405207
Recombinant IL-2	Peprotech	Cat#200-02
Recombinant IL-21	Peprotech	Cat#200-21
RNase inhibitor	Promega	Cat#N2611
Carboxymethyl cellulose	Sigma	Cat#C4888
Strep-Tactin®XT	IBA Lifesciences	Cat#2-1206-025
HEPES	Melford	Cat#34587-39108
Sodium Chloride	Honeywell	Cat#SZBF3340H
LB broth	Fisher Scientific UK	Cat#51577-51656
Mem Neaa (100X)	GIBCO	Cat#2203945
Trypsin-EDTA	GIBCO	Cat#2259288
L-Glutamine 200 mM (100X)	GIBCO	Cat#2036885
SYPROorange (5000X in DMSO)	Thermo	Cat# S6651
Isopropyl β-d-1-thiogalactopyranoside	Meridian Bioscience	Cat# BIO-37036
Kanamycin	Melford	Cat# K22000
Lysozyme	Sigma-Aldrich	Cat# L6876
Tris-base	Melford	Cat# T60040
Imidazole	Sigma-Aldrich	Cat# 56750
Triton X-100	Sigma-Aldrich	Cat# 8787
Turbonuclease	Sigma-Aldrich	Cat# T4330
RNase A	QIAGEN	Cat# 158922
NaCl	Sigma-Aldrich	Cat# S9888
MgSO4	Sigma-Aldrich	Cat# 746452
Na2HPO4	Melford	Cat# S23100
NaH2PO4	Melford	Cat# S23185

**Deposited data**		

Crystal structures of SARS-CoV-2 RBD/ **X** Fab complexes	This paper	PDB: 7BEL, 7BEI, 7BEJ, 7BEK, 7BEO, 7BEM, 7BEH, 7BEP
EM maps and structures of **X** Fab bound SARS-CoV-2 Spike	This paper	EMDB: EMD-12274, EMD-12275, EMD-12276, EMD-12277, EMD-12278, EMD-12279, EMD-12280, EMD-12281, EMD-12282, EMD-12283, EMD-12284; PDB: 7ND4, 7ND5, 7ND6, 7ND7, 7ND8, 7ND9, 7NDA, 7NDB, 7NDC, 7NDD

**Experimental models: cell lines**		

HEK293S GnTI- cells	ATCC	Cat#CRL-3022; RRID:CVCL_A785
HEK293 cells	ATCC	Cat#CRL-3216
Expi293F Cells	GIBCO,	Cat#A14527; RRID:CVCL_0063
Hamster: ExpiCHO cells	Thermo Fisher	Cat#A29133
3T3-msCD40L cells	NID AIDS Reagent Program	Cat# 2535
Vero cells	ATCC	Cat#CCL-81; RRID:CVCL_0059
Vero-furin cells	(Mukherjee et al., 2016)	N/A
MDCK-NTD	University of Oxford, NDM (A. Townsend)	N/A

**Experimental models: organisms/strains**		

Mouse: B6.Cg-Tg(K18-ACE2)2Prlmn/J	Jackson Laboratory	Cat#034860;IMSR_JAX:034860

**Oligonucleotides**		

SARS-CoV-2 N **F**: 5′-ATGCTGCAATCGTGCTACAA-3′	(Hassan et al., 2020)	N/A
SARS-CoV-2 N **R**: 5′-GACTGCCGCCTCTGCTC-3′	(Hassan et al., 2020)	N/A
SARS-CoV-2 N **Probe**: 5′-/56-FAM/TCAAGGAAC/ZEN/ AACATTGCCAA/3IABkFQ/-3′	(Hassan et al., 2020)	N/A

**Recombinant DNA**		

Vector: pHLsec	(Aricescu et al., 2006)	N/A
Vector: pOPING-ET	(Nettleship et al., 2008)	N/A
human ACE2 cDNA	Sourcebiosciences	Cat#5297380
Vector: human IgG1 heavy chain	German Cancer Research Center, Heidelberg, Germany (H. Wardemann)	N/A
Vector: human lambda light chain	German Cancer Research Center, Heidelberg, Germany (H. Wardemann)	N/A
Vector: human kappa light chain	German Cancer Research Center, Heidelberg, Germany (H. Wardemann)	N/A
Vector: Human Fab	Univeristy of Oxford	N/A
Vector: Human scFv	University of Oxford, NDM (G. Screaton)	N/A

**Software and Algorithms**		

Xia2-dials	Winter et al., 2018	https://xia2.github.io/parameters.html
PHENIX	Liebschner et al., 2019	https://www.phenix-online.org/
COOT	Emsley and Cowtan, 2004	https://www2.mrc-lmb.cam.ac.uk/personal/pemsley/coot/
PyMOL	DeLano and Bromberg	https://pymol.org/2/
Data Acquisition Software 11.1.0.11	Fortebio	https://www.sartorius.com/en/products/protein-analysis/octet-systems-software
Data Analysis Software HT 11.1.0.25	Fortebio	https://www.sartorius.com/en/products/protein-analysis/octet-systems-software
CryoSPARC v2.15.1-live	Structura Biotechnology	https://cryosparc.com/
EPU	Thermo Fisher	https://www.thermofisher.com/us/en/home/electron-microscopy/products/software-em-3d-vis/epu-software.html
SerialEM	https://bio3d.colorado.edu/SerialEM/; (Mastronarde, 2005)	N/A
Prism 8.0	GraphPad	https://www.graphpad.com/scientific-software/prism/
IBM SPSS Software 26	IBM	https://www.ibm.com/us-en/?ar=1
Biacore T200 Evaluation Software 3.1	Cytiva	https://www.cytivalifesciences.com
mabscape	This paper	https://github.com/helenginn/mabscape
		https://snapcraft.io/mabscape
Flowjo 10.7.1	BD	https://www.flowjo.com

**Other**		

X-ray data were collected at beamline I03, Diamond Light Source, under proposal IB27009 for COVID-19 rapid access	This paper	https://www.diamond.ac.uk/covid-19/for-scientists/rapid-access.html
Cryo-EM data were collected at eBIC, Diamond, under Proposal BI26983-2 for COVID-19 rapid access	This paper	https://www.diamond.ac.uk/covid-19/for-scientists/rapid-access.html
Cryo-EM grids	Cflat 2/1-200 mesh holey carbon-coated	Cat#X-301-CU200
Plunge-Freezer Vitrobot	Thermo	Cat#Vitrobot-MkIV
TALON Superflow Metal Affinity Resin	Clontech	Cat#635668
HiLoad 16/600 Superdex 200 pg	Cytiva	Cat#28-9893-35
Superdex 200 increase 10/300 GL column	Cytiva	Cat#28990944
HisTrap HP 5-ml column	Cytiva	Cat#17524802
HiTrap Heparin HT 5-ml column	Cytiva	Cat#17040703
Amine Reactive Second-Generation (AR2G) Biosensors	Fortebio	Cat#18-5092
Octet RED96e	Fortebio	https://www.sartorius.com/en/products/protein-analysis/octet-label-free-detection-systems
Buffer exchange system “QuixStand”	GE Healthcare	Cat#56-4107-78
Sonics vibra-cell vcx500 sonicator	VWR	Cat#432-0137
RT-PCR instrument for Thermofluor (Differential Scanning Fluorimetry) ex/em 492/585nm	Agilent Technologies	Cat#Mx3005P
96-well white PCR plate	4titude	Cat#4ti-0761
PCR seal	4titude	Cat#4ti-0500
Cartesian dispensing system	Genomic solutions	Cat#MIC4000
Hydra-96	Robbins Scientific	Cat#Hydra-96
96-well crystallization plate	Greiner bio-one	Cat# E20113NN
Crystallization Imaging System	Formulatrix	Cat#RI-1000

### Resource availability

#### Lead contact

Further information and requests for resources and reagents should be directed to and will be fulfilled by the Lead Contact, David I Stuart (dave@strubi.ox.ac.uk).

#### Materials availability

Recombinant proteins and antibodies generated in this study are available from the Lead Contact with a completed Materials Transfer Agreement.

#### Data and code availability

The coordinates and structure factors of the crystallographic complexes are available from the PDB with accession codes PDB: 7BEL, 7BEI, 7BEJ, 7BEK, 7BEN, 7BEO, 7BEM, 7BEH, 7BEP (see Table S5). EM maps and structure models are deposited in EMDB and PDB with accession codes EMDB: EMD-12274, EMD-12275, EMD-12276, EMD-12277, EMD-12278, EMD-12279, EMD-12280, EMD-12281, EMD-12282, EMD-12283, EMD-12284 and PDB: 7ND4, 7ND5, 7ND6, 7ND7, 7ND8, 7ND9, 7NDA, 7NDB, 7NDC, 7NDD (see Table S5). The data that support the findings of this study are available from the corresponding authors on request. Code for the competition driven mAb mapping and clustering (mabscape) is available from https://github.com/helenginn/mabscape and https://snapcraft.io/mabscape.

### Experimental model and subject details

#### Patient and blood samples

Patients were recruited from the John Radcliffe Hospital in Oxford, UK, between March and May 2020 by identification of patients hospitalised during the SARS-CoV-2 pandemic and recruited into the Sepsis Immunomics project [Oxford REC C, reference:19/SC/0296] ISARIC/WHO Clinical Characterization Protocol for Severe Emerging Infections [Oxford REC C, reference 13/SC/0149]. Time between onset of symptoms and sampling were known for all patients and if labeled as convalescent patients were sampled at least 28 days from the start of their symptoms. Written informed consent was obtained from all patients. All patients were confirmed to have tested positive for SARS-CoV-2 using the reverse transcriptase polymerase chain reaction (RT-PCR) from an upper respiratory tract (nose/throat) swab tested in accredited laboratories. The degree of severity was identified as a mild, severe or critical infection according to recommendations from the World Health Organization. Severe infection was defined for COVID-19 confirmed patients with one of the following conditions: respiratory distress with RR > 30/min; blood oxygen saturation < 93%; arterial oxygen partial pressure (PaO2) / fraction of inspired O2 (FiO2) < 300 mmHg; and critical infection was defined as respiratory failure requiring mechanical ventilation or shock; or other organ failures requiring admission to ICU. Comparator samples from healthcare workers or epidemiologically detected early clusters with confirmed SARS-CoV-2 infection who all had mild non-hospitalised disease were collected under the Gastro-intestinal illness in Oxford: COVID sub study [Sheffield REC, reference: 16/YH/0247].

Blood samples were collected and separated into plasma by centrifugation at 500 g for 10 mins. Plasma was removed from the uppermost layer and stored at −80°C. The PBMC layer was then gently suspended in the remaining plasma and RPMI media, and then isolated by Ficoll-Hypaque gradient centrifugation. All PBMC samples were stored in liquid nitrogen until use.

#### Bacterial Strains and Cell Culture

Vero (ATCC CCL-81) cells and Vero-furin cells (Mukherjee et al., 2016) were cultured at 37°C in Dulbecco’s Modified Eagle medium (DMEM) supplemented with 10% fetal bovine serum (FBS), 10 mM HEPES, and 100 U/ml of penicillin–streptomycin Spike ectodomain, human mAbs and Fabs were expressed in HEK293T cells cultured in FreeStyle 293 Expression Medium (12338018, ThermoFisher) at 37°C with 8% CO_2._ Nucleoprotein was expressed using 2-L cultures of Rosettagami2(DE3)pLysS bacteria (Novagen) in terrific broth medium containing 40 mg/L kanamycin, at 15°C for 40 hr following induction with Isopropyl β-D-1-thiogalactopyranoside (1mM final concentration, Meridian Bioscience). For ACE2 and RBD, transient expression used Expi293F cells (Thermo Fisher, Cat# A14527) grown in Expi293 Expression Medium (Thermo Fisher Cat# A1435103) in suspension with 8% CO_2_ at 30 or 37°C and shaking at 130 rpm. For production of Spike protein for structural analysis, HEKExpi293F cells (Thermo Fisher Scientific) were transfected with the construct together with a phiC31 integrase expression plasmid and grown in adhesion roller bottles with the high glucose DMEM (Sigma) with 2% FBS for 6 days at 30°C. His-tagged RBD for structural analysis was expressed in a stable HEK293S cell line cultured in DMEM (high glucose, Sigma) supplemented with 10% FBS (Invitrogen), 1 mM glutamine and 1x non-essential amino acids at 37 °C. Cells were transferred to roller bottles (Greiner) and cultured in DMEM supplemented with 2% FBS, 1 mM glutamine and 1x non-essential amino acids at 30 °C for 10 days for protein expression. For plaque assays Vero-furin cells (Mukherjee et al.,2016) were cultured at 37°C in Dulbecco’s Modified Eagle medium (DMEM) supplemented with 10% fetal bovine serum (FBS), 10 mM HEPES, and 100 U/ml of penicillin–streptomycin.

#### Viral stocks

SARS-CoV-2/human/AUS/VIC01/2020 (Caly et al., 2020) was grown in Vero (ATCC CCL-81) cells. Virus containing supernatant was spun at 2000 rpm at 4°C before being stored at −80°C. Viral titers were determined by a focus-forming assay on Vero cells. For mouse experiments, the 2019n-CoV/USA_WA1/2020 isolate of SARS-CoV-2 was obtained from the US Centers for Disease Control (CDC). Infectious stocks were propagated by inoculating Vero CCL81 cells and collecting supernatant upon observation of cytopathic effect; debris was removed by centrifugation and passage through a 0.22 μm filter. Supernatant was aliquoted and stored at −80°C.

#### Mouse experiments

Animal studies were carried out in accordance with the recommendations in the Guide for the Care and Use of Laboratory Animals of the National Institutes of Health. The protocols were approved by the Institutional Animal Care and Use Committee at the Washington University School of Medicine (assurance number A3381–01). Virus inoculations were performed under anesthesia that was induced and maintained with ketamine hydrochloride and xylazine, and all efforts were made to minimize animal suffering.

Heterozygous K18-hACE C57BL/6J mice (strain: 2B6.Cg-Tg(K18-ACE2)2Prlmn/J) were obtained from The Jackson Laboratory. Seven to eight-week-old male and female animals were inoculated with 10^3^ PFU of SARS-CoV-2 via intranasal administration.

### Method details

#### Trimeric spike of SARS-CoV-2

To construct the expression plasmids for SARS-CoV-2 spike protein, a gene encoding residues 1−1208 of the spike ectodomain with a mutation at the furin cleavage site (residues 682-685) from RRAR to GSAS, proline substitutions at residues 986 and 987, followed by the T4 fibritin trimerization domain, a HRV3C protease cleavage site, a twin Strep Tag and an 8XHisTag, was synthesized and optimized for mammalian expression (Wrapp et al., 2020). An optimized coding sequence was cloned into the mammalian expression vector pHLsec.

#### Trimeric spike of SARS-CoV, MERS-CoV, OC63-CoV, HKU1-CoV, 229E-Cov, NL63-CoV

Expression plasmids were constructed using synthetic fragments coding for human codon-optimized Spike glycoprotein sequences from CoV-229E (GenBank accession number NC_002645.1; amino acids 1–1113), CoV-HKU1 (GenBank accession number NC_006577.2; amino acids 1-1300), CoV-NL63 (GenBank accession number NC_005831.2; amino acids 1–1289), CoV-OC43 (GenBank accession number NC_006213.1; amino acids 1–1297), CoV-MERS (GenBank accession number AFS88936.1; amino acids 1-1291) (Zhao et al., 2013), CoV-SARS1 (GenBank accession number AY27874; amino acids 11-1195) (Simmons et al., 2004) and CoV-SARS2 (GenBank accession number MN908947; amino acids 1-1208). Fragments were cloned in pHLsec vectors downstream of the chicken β-actin/rabbit β-globin hybrid promoter and followed by a T4 fibritin trimerization domain, an HRV 3C cleavage site, a His-8 tag and a Twin-Strep-tag at the C terminus as previously reported by Wrapp et al. (2020).

Mutations coding for stabilizing proline residues and to eliminate putative furin cleavage sites were inserted in each sequence as follows: For CoV-229E, TI > PP (aa 871-872); for CoV-HKU1, RRKR > GSAS (aa 756-759) and AL > PP (aa 1071-1072); for CoV-NL63, RRSR > GSAS (aa 754-757) and SI > PP (aa 1052-1053); for CoV-OC43, AL > PP (aa 1070-1071); for CoV-MERS, RSVG > ASVG (aa 748), RSAR > GSAS (aa 884-887) and VL > PP 1060-1061; for CoV-SARS1, KV > PP (aa 968-969); for CoV-SARS2, RRAR > GSAS (aa 682-685) and KV > PP (aa 986-987). All sequences were verified by DNA sequencing.

DNA plasmids encoding the Strep-Tag-tagged spike proteins were transfected into HEK293T cells cultured in FreeStyle 293 Expression Medium (12338018, ThermoFisher) by PEI-mediated transfection (MW: 25,000; branched: Sigma-Aldrich 408727) and incubated at 37 °C for 7 days. Supernatants were then collected and cleared by centrifugation followed by filtration. CoV Spike protein trimers were affinity-purified using the Strep-Tactin®XT purification system (IBA Lifesciences) according to the instructions of the manufacturer. In the case of CoV-229E and CoV-NL63, the spike proteins were further purified by SEC (Superose 6 increase 30/100 GL column, GE Life Sciences; elution buffer: Tris 20mM, NaCl 150mM, pH 7) to remove aggregates. The purity of the proteins was assessed by reducing (10% β-mercaptoethanol (β-ME)) and non-reducing sodium dodecyl sulfate polyacrylamide gel electrophoresis (SDS-PAGE) (~3 μg of protein). Purified proteins were concentrated in PBS, quantified by spectrophotometry, sterilized by filtration (Spin-X tube filter; 8160; Costar) and kept at −80 °C until use.

#### Nucleoprotein (NP)

The native SARS-CoV-2 nucleoprotein gene was cloned into a pET28a(+) vector (Novagen) downstream of the coding sequence for an N-terminal hexa-histidine tag and 3C-protease cleavage site (a gift from Fred Antson). Expression was carried out using 2-L cultures of Rosettagami2(DE3)pLysS bacteria (Novagen) in terrific broth medium containing 40 mg/L kanamycin, at 15°C for 40 hr following induction with Isopropyl β-D-1-thiogalactopyranoside (1mM final concentration, Meridian Bioscience). Upon centrifugation (10,000 x g; 20 minutes, 4°C), pellets were resuspended in 60 mL H_2_O containing 10 mg/ml lysozyme (Sigma). After adding 70 mL buffer S (200 mM Tris pH 8.0, 2.5 M NaCl, 60 mM imidazole, 4 mM MgSO4, 0.2% Triton X-100) the suspension was sonicated (40% amplitude, 10 s on-10 s off cycles, 20 min, 4°C). Turbonuclease (3,000 units, Sigma) and RNaseA (500 units, QIAGEN) were added, and the solution was clarified (20,000 x g, 30 min, 4°C) before purification over a 5 mL HisTrap column (Cytiva), using a 20 mM to 1 M imidazole gradient in 25 mM Tris pH 8.0, 1.5 M NaCl. Nucleoprotein-containing fractions were further purified over a Superdex 200 Increase 10/300 GL column (Cytiva) using a 25 mM Tris pH 8.0, 1 M NaCl running buffer, followed by buffer exchange into phosphate-buffered saline (PBS, Sigma) using PD-10 columns (Cytiva), and heparin-affinity chromatography using a 5 mL HiTrap heparin HT column (Cytiva) and a 0.15 - 1 M NaCl gradient in 40 mM sodium phosphate pH 7.4.

#### Depletion of anti-RBD antibodies from plasma samples

Nickel charged agarose beads (nickel-nitrilotriacetic acid [Ni-NTA]; QIAGEN) were washed 3 times in PBS and then incubated overnight, rotating at 4°C, with His-tagged RBD. Twenty micrograms of protein were added for every 50 μL of beads used in a final incubation volume, twice the bead volume. Beads incubated in the absence of RBD antigen were used as a beads-only, mock control. The beads were then washed 3 times with PBS and precleared for 2h at RT with a pooled SARS-CoV-2 negative plasma at a dilution of 1 in 100 in an incubation volume 2 times the bead volume. Beads were then washed 3 times in PBS and incubated with the human plasma samples of interest at a dilution of 1:50 in PBS^+^, PBS containing an additional 150 mM NaCl and 20 mM imidazole, for 2h at 4°C (50 μL beads per 200 μL sample). The remaining depleted samples were collected, filter sterilized, and tested for complete depletion by RBD direct ELISA.

#### ACE2 and RBD

Constructs are as described in Huo et al. (2020) and production was as described in Zhou et al. (2020).

#### Isolation of human monoclonal antibodies from peripheral B cells by memory B cell stimulation

To generate human monoclonal antibodies from peripheral blood B cells, CD22+ B cells were isolated from PBMCs using CD22 Microbeads (130-046-401; Miltenyi Biotec). Pre-enriched B cells were stained with anti-IgM-APC, IgA-FITC and IgD-FITC. Double negative memory B cells (IgM-,IgA-/D-cells) were sorted by FACS and plated on 384-well plates at a density of 4 B cells per well. Cells were stimulated to proliferate and produce IgG by culturing with irradiated 3T3-msCD40L feeder cells (12535; NID AIDS Reagent Program), 100 U/ml IL-2 (200-02; Peprotech) and 50 ng/ml IL-21 (200-21; Peprotech) for 13-14 days. Supernatants were harvested from each well and screened for SARS-CoV-2 binding specificity by ELISA. Lysis buffer was added to positive wells containing SARS-CoV-2-specific B cells and immediately stored at −80°C for future use in Ig gene amplification and cloning.

#### Isolation of Spike and RBD-specific single B cells by FACS

To isolate Spike and RBD-specific B cells, PBMCs were sequentially stained with LIVE/DEAD Fixable Aqua dye (Invitrogen) followed by recombinant trimeric spike-twin-Strep or RBD-biotin. Cells were then stained with antibody cocktail consisting of CD3-FITC, CD14-FITC, CD56-FITC, CD16-FITC, IgM-FITC, IgA-FITC, IgD-FITC, IgG-BV786, CD19-BUV395 and Strep-MAB-DY549 (iba) or streptavidin-APC (Biolegend) to probe the Strep tag of spike or biotin of RBD. Spike or RBD-specific single B cells were gated as CD19+, IgG+, CD3-, CD14-, CD56-, CD16-, IgM-, IgA-, IgD-, Spike+ or RBD+ and sorted into each well of 96-well PCR plates containing RNase inhibitor (N2611; Promega). Plates were centrifuged briefly and frozen on dry ice before storage at −80°C for future use in Ig gene amplification and cloning.

#### Cloning and expression of SARS CoV2-specific human mAbs

Genes encoding Ig VH, Ig Vκ and Vλ from positive wells were recovered using RT-PCR (210210; QIAGEN). Nested PCR (203205; QIAGEN) was then performed to amplify genes encoding γ-chain, λ-chain and κ-chain with ‘cocktails’ of primers specific for human IgG. PCR products of genes encoding heavy and light chains were joined with the expression vector for human IgG1 or immunoglobulin κ-chain or λ-chain (gifts from H. Wardemann) by Gibson assembly. For the expression of antibodies, plasmids encoding heavy and light chains were co-transfected into the 293T cell line by the polyethylenimine method (408727; Sigma), and antibody-containing supernatants were harvested for further characterization.

#### Construction of Fab expression plasmids

Heavy chain expression plasmids of specific antibodies were used as templates to amplify the first fragment, heavy chain vector include the variable region and CH1 until Kabat amino acid number 233. The second fragment of thrombin cleavage site and twin-Strep-tag with overlapping ends to the first fragment were amplified. The two fragments were ligated by Gibson assembly to make the Fab heavy chain expression plasmid.

#### Construction of scFv antibody plasmid

Heavy chain and light chain expression plasmids of specific antibodies were used as a template to amplify variable region gene of heavy and light chain respectively. First, heavy chain gene products having the AgeI–SalII restriction enzyme sites were cloned into a scFv vector which is a modified human IgG expression vector which has a linker between the H chain and L chain genes followed by a thrombin cleavage site and twin-Strep-tags. Light chain gene products having NheI-NotI restriction enzyme site were cloned into scFv vector containing the heavy chain gene insert to produce scFv expression plasmids.

#### Fab and scFv production and purification

Protein production was done in HEK293T cells by transient transfection with polyethylenimine in FreeStyle 293 medium. For Fab antibody production, Fab heavy chain expression plasmids were co-transfected with the corresponding light chain. For scFv antibody production, scFv expression plasmid of specific antibody was used for transfection. After 5 days of culture at 37°C and 5% CO2, culture supernatant was harvested and filtered using a 0.22 mm polyethersulfone (PES) filter. Fab and scFv antibody were purified by Strep-Tactin affinity chromatography (IBA lifescience) according to the Strep-Tactin XT manual.

#### Determination of plasma and antibody binding to recombinant protein by ELISA

MAXISORP immunoplates (442404; NUNC) were coated with 0.125 μg of StrepMAB-Classic (2-1507-001;iba) at 4°C overnight and blocked with 2% skimmed milk in PBS (for plasma) or 2% BSA in PBS (for mAbs) for 1 h, plates were incubated with 50 μL of 10 μg/mL double strep-tag recombinant spike of SARS-CoV-2, SARS-CoV, MERS-CoV, OC43-CoV, HKU1-CoV, 229E-CoV and NL43-CoV. After one hour, 50 μL of serially diluted plasma or mAbs was added, followed by ALP-conjugated anti-human IgG (A9544; Sigma) at 1:10,000 dilution. The reaction was developed by the addition of PNPP substrate and stopped with NaOH. The absorbance was measured at 405nm. To determine the binding to SARS-CoV-2 RBD, SARS-CoV-2 NP, SARS-CoV-2 spike S1 (40591-V08H; Sino Biological Inc) and SARS-CoV-2 spike S2 (40590-V08B; Sino Biological Inc), immunoplates were coated with 0.125 μg of Tetra-His antibody (34670; QIAGEN) followed by 5 μg/mL of His-tag recombinant SARS-CoV-2 RBD, SARS-CoV-2 NP, SARS-CoV-2 spike S1 and SARS-CoV-2 spike S2. The plasma endpoint titers (EPTs) were defined as reciprocal plasma dilutions that corresponded to two times the average OD values obtained with mock. EC_50_ of mAbs were evaluated using non-linear regression (curve-fit), GraphPad Prism 8 software.

#### Whole Virus ELISA

To determine the binding affinity of antibody to SARS-CoV-2 virus, virus was captured onto plates coated with mouse anti-SARS-CoV-2 spike (mAb31 with murine Fc) and then incubated with serial dilutions of SARS-CoV-2-specific human mAbs (full length IgG or Fab) followed by ALP-conjugated anti-human IgG (A8542, Sigma). The reaction was developed with PNPP substrate and stopped with NaOH. The absorbance was measured at 405 nm.

Results are expressed as the percentage of total binding, with 100% binding determined from the Ab concentration that gave maximum absorbance. GraphPad PRISM software was used to perform nonlinear regression curve-fitting analyses of binding data to estimate dissociation constants (K_d_). Percent occupancy at IC_50_ was determined using the following formula: Percent occupancy = BMax^∗^ [Ab]/(Kd+[Ab]), where the BMax is percent maximal binding, [Ab] is the concentration of Ab required to reach 50% FRNT and Kd is the concentration of Ab required to reach half-maximal binding.

#### Focus Reduction Neutralization Assay (FRNT)

The neutralization potential of Ab was measured using a Focus Reduction Neutralization Test (FRNT), where the reduction in the number of the infected foci is compared to a no antibody negative control well. Briefly, serially diluted Ab was mixed with authentic SARS-CoV-2/human/AUS/VIC01/2020 (Caly et al., 2020) and incubated for 1 hr at 37°C. The mixtures were then transferred to Vero cell monolayers and incubated for 2 hr followed by the addition of 1.5% semi-solid carboxymethyl cellulose *(*CMC*)* overlay medium to each well to limit virus diffusion. A focus forming assay was then performed by staining Vero cells with human anti-NP mAb (mAb206) followed by peroxidase-conjugated goat anti-human IgG (A0170; Sigma). Finally, the foci (infected cells) were visualized by adding TrueBlue Peroxidase Substrate. The percentage of focus reduction was calculated and IC_50_ was determined using the probit program from the SPSS package.

#### NTD Binding Assay

MAbs were screened for binding to MDCK-SIAT1 cells expressing the N-terminal domain (NTD) of SARS-CoV-2 spike glycoprotein (MDCK-NTD). MDCK-NTD was created by stably transfecting MDCK-SIAT1 cells (ECACC 05071502) (Matrosovich et al., 2003) with cDNA encoding the SARS-CoV-2 NTD (amino acids VNLT…TLKS) fused to the transmembrane domain of haemagglutinin H7 (A/HongKong/125/2017) (EPI977395) at the C terminus for surface expression using a second-generation lentiviral vector system. NTD expressing cells were FACS sorted using the FD7C mAb (Huang et al., 2020). In brief, MDCK-NTD cells were seeded at 3 × 10^4^ per well in flat-bottomed 96-well plates (TPP) in high glucose DMEM containing 10% fetal bovine serum (FBS) at 37°C overnight. The medium was then removed and washed with 2% FBS in PBS (PBS/2% FBS) twice. 10 μg/ml of mAbs supernatants from transfected 293T cells were added (50 μl per well) and incubated at room temperature for 1 h. A second antibody Goat anti-human IgG Fc specific-FITC (F9512, Sigma-Aldrich) diluted 1:300 in PBS/2% FBS was then added (50 μl per well) and incubated for another 1 h at room temperature. After washing twice with PBS, the wells were fixed with 1% formaldehyde in PBS. The binding antibodies were detected by fluorescence intensities using a Clariostar plate reader (BMG, Labtech).

#### ELISA based ACE2 binding inhibition assay

For the ACE2 competition ELISA, 250 ng of ACE2 protein was immobilized to a MAXIXORP immunoplate and the plates were blocked with 2% BSA in PBS. In the meantime, serially diluted Ab was mixed with recombinant RBD-mFc (40592-V05H; Sino Biological) and incubated for 1 h at 37°C. The mixtures were then transferred to the ACE2 coated plates and incubated for 1 h followed by goat anti-mouse IgG Fc-AP (Invitrogen #A16093) at 1:2000 dilution. The reaction was developed by the addition of PNPP substrate and stopped with NaOH. The absorbance was measured at 405 nm. The ACE2/RBD binding inhibition rate was calculated by comparing to antibody-free control well. IC_50_ were determined using the probit program from the SPSS package.

#### Spike protein production for structural analysis

The stable cell line generation vector pNeoSec was used for cloning of the SARS-Cov2 Spike ectodomain comprising amino acids 27-1208 with mutations of the furin cleavage site (RRAR > GSAS at residues 682-685) and the PP (KV > PP at residues 986-987). At the N terminus, there is a twin StrepII tag and at the C terminus fused with a T4 fibritin trimerisation domain, an HRV 3C cleavage site and a His-8 tag. The human embryonic kidney (HEK) Expi293F cells (Thermo Fisher Scientific) were transfected with the construct together with a phiC31 integrase expression plasmid as described earlier (Zhao et al., 2014). The polyclonal G418 resistant (1 mg/ml) cell population were used for protein production. Expi293F cells were grown in adhesion in roller bottles with the high glucose DMEM (Sigma) with 2% FBS for 6 days at 30°C. The soluble spike protein was captured from the dialysed conditional media with prepacked 5 mL Columns of HisTrap excel (GE Healthcare Life Sciences). The protein was eluted in 300 mM imidazole containing phosphate-buffered saline (PBS) after a 20 mM imidazole PBS wishing step. The protein was further purified with a 16/600 Superdex 200 size exclusion chromatography with an acidic buffer (20 mM Acetate, 150 mM NaCl, pH 4.6) for the low pH Spike incubations, or a neutral buffer (2 mM Tris, 150 mM NaCl, pH 7.5).

#### Production of RBD for structural analysis

Stable HEK293S cell line expressing His-tagged RBD was cultured in DMEM (high glucose, Sigma) supplemented with 10% FBS (Invitrogen), 1 mM glutamine and 1x non-essential amino acids at 37 °C. Cells were transferred to roller bottles (Greiner) and cultured in DMEM supplemented with 2% FBS, 1 mM glutamine and 1x non-essential amino acids at 30 °C for 10 days for protein expression. For protein purification, the dialyzed media was passed through a 5 mL HisTrap Nickel column (GE Healthcare). The column was washed with buffer 20 mM Tris pH 7.4, 200 mM NaCl, 30 mM imidazole and RBD was eluted using buffer 20 mM Tris pH 7.4, 200 mM NaCl, 300 mM imidazole. A volume of 30 μL endoglycosidase H1 (~1 mg ml^−1^) was added to ~30 mg RBD and incubated at room temperature for 2 h. Then the sample was further purified with a Superdex 75 HiLoad 16/600 gel filtration column (GE Healthcare) using 10 mM HEPES pH 7.4, 150 mM NaCl. Purified RBD was concentrated using a 10-kDa ultra centrifugal filter (Amicon) to 10.6 mg ml^−1^ and stored at −80°C.

#### Preparation of Fabs from IgGs

Fab fragments were digested from purified IgGs with papain using a Pierce Fab Preparation Kit (Thermo Fisher), following the manufacturer’s protocol.

#### Physical assays

Thermal stability was assessed using Thermofluor (DSF). Briefly, 3 μg of the Ab preparation was used in a 50 μl reaction containing 10 mM HEPES pH 7.5, 100 mM NaCl, 3X SYPROorange (Thermo Fisher). Samples were heated from 25-97°C in a RT-PCR machine (Agilent MX3005p) and the fluorescence monitored at 25°C after every 1°C of heating. Melting temperatures (Tm) were calculated by fitting of a 5-parameter sigmoid curve using the JTSA software (P. Bond, https://paulsbond.co.uk/jtsa). Polydispersity was assessed by DLS using 10 μg of the Ab preparation in an UNCLE instrument (Unchained Labs). Freeze thaw experiments on 4 of the mAbs were performed with material at 1 mg/ml by flash-freezing using LN2, thawing and centrifuging an aliquot (10 minutes at 20000 g) before measuring the absorbance at 280nm of the soluble fraction.

#### Crystallization

Purified RBD was combined separately with Strep-tagged Fab150, Fab58, scFv269 and Fab316 in a 1:1 molar ratio, with final concentrations of 13.2, 9.4, 12.7 and 13.0 mg ml^-1^, separately. RBD was combined with Fab45 and Strep-tagged Fab88, Fab75 and Fab253, and Fab 75 and Strep-tagged chimeric Fab 253H55L in a 1:1:1 molar ratio all with a final concentration of 7 mg ml^−1^, separately. Glycosylated RBD was combined with Fab S309 (Pinto et al., 2020) and Fab384 in a 1:1:1 molar ratio with a final concentration of 8 mg ml^−1^. These complexes were separately incubated at room temperature for 30 min. Initial screening of crystals was set up in Crystalquick 96-well X plates (Greiner Bio-One) with a Cartesian Robot using the nanoliter sitting-drop vapor-diffusion method, with 100 nL of protein plus 100 nL of reservoir in each drop, as previously described (Walter et al., 2003). Good crystals of RBD-150 complex were formed in Molecular Dimensions Morpheus condition C2, containing 0.09 M NPS (nitrate, phosphate and sulfate), 0.1 M MES/imidazole pH 6.5, 10% (w/v) PEG 8000 and 20% (v/v) ethylene glycol and crystals also formed in Hampton Research PEGRx condition D11, containing 0.1 M imidazole pH 7.0 and 12% (w/v) PEG 20000. Some good crystals of RBD-158 were obtained from Index condition C01, containing 3.5 M NaCOOH pH 7.0, while some crystals were formed in Proplex condition C1, containing 0.15 M (NH4)_2_SO_4_, 0.1 M Tris pH 8.0 and 15% (w/v) PEG 4000 and further optimized in 0.15 M (NH4)_2_SO_4_, 0.1 M Tris pH 7.6 and 14.6% (w/v) PEG 4000. Crystals of RBD-scFv269 complexed were obtained from Index condition F01, containing 0.2 M Proline, 0.1 M HEPES pH 7.5 and 10% (w/v) PEG 3350. Good crystals for the RBD-316 complex were obtained from Index condition G10, containing 0.2 M MgCl2, 0.1 M bis-Tris pH 5.5 and 25% (w/v) PEG 3350. Crystals of RBD-45-88 complex were obtained from PEGRx condition G12, containing 10% (v/v) 2-Propanol, 0.1 M Sodium acetate trihydrate pH 4.0, 22% (w/v) PEG 6000. Crystals of RBD-75-253 complex were obtained from PEGRx condition D8, containing 0.1 M BIS-TRIS pH 6.5, 16% (w/v) PEG 10000. Crystals of RBD-75-253H55L were obtained from Index condition F5, containing 0.1 M ammonium acetate, 0.1 M bis-Tris pH 5.5 and 17% (w/v) PEG 10000. For the RBD-S309-384 ternary complex, good crystals were obtained from Morpheus condition H1, containing 0.1 M amino acids (Glu, Ala, Gly, Lys, Ser), 0.1 M MES/imidazole/ pH 6.5, 10% (w/v) PEG 20000 and 20% (w/v) PEG MME 550.

#### X-ray data collection, structure determination and refinement

Crystals were soaked in a solution containing 25% glycerol and 75% reservoir solution for a few seconds and then mounted in loops and frozen in liquid nitrogen prior to data collection. Diffraction data were collected at 100 K at beamline I03 of Diamond Light Source, UK. Diffraction images of 0.1° rotation were recorded on an Eiger2 XE 16M detector with exposure time ranging from 0.004 to 0.01 s per frame, beam size 80 × 20 μm and 100% beam transmission. Data were indexed, integrated and scaled with the automated data processing program Xia2-dials or Xia2-3dii (Winter, 2010; Winter et al., 2018). For RBD-158 crystal form 2, RBD-316 and the ternary complexes of RBD88-45, RBD-253H55L and RBD-384-S309 datasets of 360° were collected from a single frozen crystal each, and 720° of data from 2 crystals for RBD-150, RBD-scFv269, RBD-158 crystal form 1 and RBD-253-75.

The structures were determined by molecular replacement with PHASER (Liebschner et al., 2019) using search models of the RBD, VhVl and ChCl domains of a closely related Fab in sequence for each complex. Sequence corrections to the target Fabs from the search models and model rebuilding were done with COOT (Emsley and Cowtan, 2004). All the structures were refined with PHENIX (Liebschner et al., 2019) resulting in good R-factors and stereochemistry for most of the structures except for RBD-88-45 and RBD-53-75 in each of which there is presence of translational NCS with vectors (−0.003 0.502 0.489) and (0.044, 0, 0.5) and can only be refined to R_work_/R_free_ of 0.250/0.285 and 0.242/0.284 to 2.53 Å and 2.50 Å, respectively. The ChCl domains of Fab 88 in the RBD-88-45 complex are disordered. Data collection and structure refinement statistics are given in Table S5.

#### Cryo-EM Grid Preparation

For all Fab or IgG-Spike complexes, a 3 μL aliquot of S ~0.6 μm (determined by OD) with Fab (1:6 molar ratio) was prepared, aspirated and almost immediately applied to a freshly glow-discharged Cu support Cflat 2/1-200 mesh holey carbon-coated grid (high intensity, 20 s, Plasma Cleaner PDC-002-CE, Harrick Plasma). Excess liquid was removed by blotting for 5-5.5 s with a force of −1 using vitrobot filter paper (grade 595, Ted Pella Inc.) at 4.5°C, 100% reported humidity before plunge freezing into liquid ethane using a Vitrobot Mark IV (Thermo Fisher).

#### Cryo-EM Data collection and processing

##### 40, 253H55L and 253H165L spike complexes:

For sample-specific details, refer to Table S6.

Movies were collected in compressed tiff format on a Titan Krios G2 (Thermo Fisher) operating at 300 kV with a K3 detector (Gatan) in super resolution counting mode using a custom version of EPU 2.5 (Thermo Fisher). A defocus range of 0.8-2.6 μm was applied with a nominal magnification of x105,000, corresponding to a calibrated pixel size of 0.83 Å/pixel and with a total dose of 43-47 e/ Å^2^, see Table S6.

Two-times binned movies were then motion corrected and aligned on the fly using Relion(3.1) scheduler (Zivanov et al., 2018) with a 5 × 5 patch based alignment. CTF-estimation of full-frame non-weighted micrographs was performed with the GCTF (1.06) (Zhang, 2016) module in cryoSPARC(v2.14.1-live) (Punjani et al., 2017).

##### 88, 150, 158, 159IgG, 316 and 384 spike complexes:

Data for 88, 150, 158 were collected using a Titan Krios G2 (Thermo Fischer) operating at 300 kV with a K2 camera and a GIF Quantum energy filter (Gatan) with a 30 eV slit. For 159 (IgG), 384 and 316, data were collected as for 88, 150 and 158, except using a 20 keV slit. Rapid multi-shot data acquisition was set up using custom scripts with SerialEM (version 3.8.0 beta) (Mastronarde, 2005) at a nominal magnification of 165 kX, corresponding to a calibrated pixel size of 0.82 Å per pixel. A defocus range of −0.8 μm to −2.6 μm was used with a total dose of ~45-57 e^-^/Å^2^ applied across 40 frames. Motion and CTF correction of raw movies was performed on the fly using cryoSPARC live patch-motion and patch-CTF correction (Punjani et al., 2017).

##### 40, 253H55L, 253H165L, 88, 150, 158, 159 IgG, 316 and 384 complexes:

Poor-quality images were discarded after manual inspection of CTF and motion estimations. Particles were then blob picked in cryoSPARC (Punjani et al., 2017) and initially extracted with four times binning. After inspection of 2D classes, classes of interest were selected to generate templates for complete particle picking. Binned particles were then subjected to one to three rounds of reference free 2D classification followed by 3D classification with an ab-initio derived model before further refinement and unbinning.

For both 150 and 158, two data separate data collections were set up on the same grid, and refined particle sets from each collection were separated by exposure groups before being combined. For 150, a total of 77,265 exposure-group split particles were initially combined (51,554 from 4726 movies and 25,711 from 2079 movies), re-classified into five classes, and the two best classes (42,655 particles) subjected to further non-uniform refinement, with obvious density for Fab bound to one RBD in an ‘up’ conformation. Notably, discarded classes included a high proportion of undecorated S (28,463 particles, 4.4 Å reported resolution at GSFSC = 0.143, −43 Å^2^ B-factor).

Classification using heterogeneous refinement in cryoSPARC was found to be generally poor, and, instead, 3D variability analysis was employed to try to better resolve full spike-Fab structures. Local refinements were also performed with masks focused around the Fab/RBD region (not reported here), but maps were still insufficient to clearly build a model at the RBD/Fab interface and far inferior to the crystallographic maps. 3D variability analysis was found to be essential for isolating the RBD up and RBD down conformations for 159-IgG. Results from this are presented for 159-IgG and 384. Briefly, data were separated into eight clusters using the 3D variability analysis module with a 6 Å resolution filter and a mask around the RBD/Fab region. Masks were generated by initially rigid body fitting a model of the spike and a Fab into a refined map in Chimera before selecting an area of the model including the RBD and fab and using the ‘color zone’ module to crop out this desired part of the map. The resulting map was smoothed with a Gaussian filter (Pettersen et al., 2004), converted into a mask format using Relion3.1 ‘Mask Create’ before import into cryoSPARC. Resolution estimates quoted in the Table S6 were taken from Gold standard-FSC (FSC = 0.143) reported in the local resolution module in cryoSPARC (Punjani et al., 2017).

#### Competition assay of antibodies

Competition assay of anti-RBD antibodies was performed on a Fortebio Octet RED96e machine with Fortebio Anti-HIS (HIS2) Biosensors. 2 μg ml^−1^ of His-tagged RBD dissolved in the running buffer (10 mM HEPES, pH 7.4 and 150 mM NaCl) was used as the ligand and was first immobilized onto the biosensors. The biosensors were then washed in the running buffer to remove unbound RBD. Each biosensor was dipped into different saturating antibodies (Ab1) to saturate the bound RBD, except one biosensor was into the running buffer in this step, acting as the reference. The concentration of saturating antibodies used was 15 μg ml^−1^. Higher concentrations were applied if 15 μg ml^−1^ was not enough to obtain saturating. Then all biosensors were washed with the running buffer again and dipped into wells containing the same competing antibody (Ab2). The concentration of competing antibodies used was 5 μg ml^−1^. The y axis values of signals of different saturating antibodies in this step were divided by the value of the reference channel to get ratio results of different Ab1-Ab2 pairs. Ratio result close to 0 indicated total competition while 1 indicated no competition. In total, 50 IgGs and 4 Fabs (Fabs 40, EY6A [Zhou et al., 2020], FD5D (unpublished) and S309 [Pinto et al., 2020]) were used as the saturating antibodies and 80 IgGs as the competing antibodies.

#### Competition mapping of antibodies

##### Gross binning of antibodies

Competition values were prepared for cluster analysis and binning by capping all competition values between 0 and 1. Competition values between antibodies *i* and *j* were averaged with the competition value for *j* and *i* when both were available. *Cluster4x* (Ginn, 2020) was used to cluster antibodies into three distinct groups using single value decomposition on the matrix of competition values.

##### Preparation of RBD surface and mesh

A surface of the receptor-binding domain was generated in PyMOL (The PyMOL Molecular Graphics System, Version 1.2r3pre, Schrödinger, LLC) from chain E of PDB code 6YLA. A mesh was generated and iteratively contracted and restrained to the surface of the RBD to provide a smoother surface on which to direct antibody refinement, reducing intricate surface features which could lead to unrealistic exploration of local minima.

##### Fixing positions of antibodies with known structure

In order to provide an objective position for those antibodies of known structure (FD5D (unpublished), EY6A (Zhou et al., 2020), S309 (Pinto et al., 2020) and mAb 40), to reflect the occluded region, all non-hydrogen antibody atoms were found within 20 Å of any RBD atom, and likewise all RBD atoms within 20 Å of an antibody atom. From each group, the atoms with the lowest sum-of-square-lengths from all other members were identified and the midpoint of these two atoms was locked to the nearest vertex on the mesh. Solvent molecules were ignored, but in the case of S309, the glycan cofactor was included in the set of antibody atoms.

##### The target function

On an evaluation of the target function, either all unique pairs of antibodies were considered (all-pairs), or only unique pairs where one of the antibodies was fixed (fixed-pairs), depending on the stage of the minimization protocol. Competition levels were estimated for each pair of antibodies as described by f(x) in Equation 1

$$f\left(x\right)=\frac{e^{\frac{r-d}{2}}}{1+e^{\frac{r-d}{2}}}$$

where *r* is the working radius of the antibody, set to 11 Å, accounting for the approximate antibody radius. The distance between the pair of antibodies at a given evaluation of the function is given by *d* in Angstroms. The target function was the sum of squared differences between the competition estimation and the competition value from SPR data.

##### Obtaining a self-consistent set of refined antibody positions

Minimization was carried out globally by 1000 macrocycles of Monte Carlo-esque sampling using LBFGS refinement. A random starting position for each antibody was generated by randomly assigning a starting vertex on the RBD mesh and the target function minimized for 20 cycles considering data points for pairs with at least one fixed antibody, followed by 40 cycles for all data points. Between each cycle, antibody positions were locked onto the nearest mesh vertex. Depending on the starting positions of antibodies, results were a mixture of well-refined and poorly refined solutions. Results were ordered in ascending target function scores. Positions of antibodies for each result was passed into cluster4x as dummy C-alpha positions (Ginn, 2020). A clear self-consistent solution was enriched in lower target function scores and separated using cluster4x for further analysis. The average position for each antibody was chosen as the sampled position which had the lowest average square distance to very other sampled position, and the RMSD calculated from all contributing antibody positions.

#### Measurement of viral burden (*in vivo* experiments)

Tissues were weighed and homogenized with zirconia beads in a MagNA Lyser instrument (Roche Life Science) in 1000 μL of DMEM supplemented to contain 2% heat-inactivated FBS. Tissue homogenates were clarified by centrifugation at 10,000 rpm for 5 min and stored at −80°C. RNA was extracted using the MagMax mirVana Total RNA isolation kit (Thermo Scientific) on a Kingfisher Flex extraction robot (Thermo Scientific). RNA was reverse transcribed and amplified using the TaqMan RNA-to-CT 1-Step Kit (ThermoFisher). Reverse transcription was carried out at 48°C for 15 min followed by 2 min at 95°C. Amplification was accomplished over 50 cycles as follows: 95°C for 15 s and 60°C for 1 min. Copies of SARS-CoV-2 N gene RNA in samples were determined using a previously published assay (PubMed ID 32553273). Briefly, a TaqMan assay was designed to target a highly conserved region of the N gene (Forward primer: ATGCTGCAATCGTGCTACAA; Reverse primer: GACTGCCGCCTCTGCTC; Probe: /56-FAM/TCAAGGAAC/ZEN/AACATTGCCAA/3IABkFQ/). This region was included in an RNA standard to allow for copy number determination. The reaction mixture contained final concentrations of primers and probe of 500 and 100 nM, respectively.

#### Plaque assay

Vero-furin cells (Mukherjee et al., 2016) were seeded at a density of 2.5 × 10^5^ cells per well in flat-bottom 12-well tissue culture plates. The following day, medium was removed and replaced with 200 μL of 10-fold serial dilutions of the material to be titrated, diluted in DMEM+2% FBS. After incubation for 1 h at 37°C, 1 mL of methylcellulose overlay was added. Plates were incubated for 72 h, then fixed with 4% paraformaldehyde (final concentration) in phosphate-buffered saline for 20 min. Plates were stained with 0.05% (w/v) crystal violet in 20% methanol and washed twice with distilled, deionized water prior to plaque enumeration.

#### Affinity determination using biolayer interferometry

Octet RED 96e (ForteBio) was used to determine the binding affinities of antibodies with RBD or spike. Anti-RBD IgGs were immobilized onto AR2G biosensors (ForteBio) while RBD was used as the analyte with serial dilutions. For IgG159, spike was immobilised onto AR2G biosensors with IgG159 acting as the analyte with serial dilutions. Kd values were calculated using Data Analysis HT 11.1 (ForteBio) with a 1:1 global fitting model.

### Quantification and statistical analysis

ELISA K_d_ and EC_50_ values were estimated using nonlinear regression curve-fitting analyses of binding data, Prism Version 8 software (GraphPad). The percentage of focus reduction was calculated and IC_50_ was determined using the probit program from the SPSS package. IC_50_ were determined using the probit program from the SPSS package. Neutralization potencies (IC_50_) between 2 groups of antibodies were compared using two-tailed Mann–Whitney U test, Prism Version 8 software (GraphPad). BLI Kd values were calculated using Data Analysis HT 11.1 (ForteBio) with a 1:1 global fitting model. Statistical analysis of weight change and viral burden *in vivo* were determined by two-way ANOVA with Sidak’s post-test and Kruskal-Wallis test with Dunn’s post-test, Prism Version 8 software (GraphPad), respectively.
