# Supplementary material for: The antigenic anatomy of SARS-CoV-2 receptor binding domain
Source: Cell. 2021 Apr 15;184(8):2183–2200.e22. doi: 10.1016/j.cell.2021.02.032 (PMC7891125; doi:10.1016/j.cell.2021.02.032)
Supplement: Document S1. Tables S1–S7 [file mmc1.pdf]

Cell, Volume *184*

Supplemental information

The antigenic anatomy of SARS-CoV-2 receptor binding domain

Wanwisa Dejnirattisai, Daming Zhou, Helen M. Ginn, Helen M.E. Duyvesteyn, Piyada Supasa, James Brett Case, Yuguang Zhao, Thomas S. Walter, Alexander J. Mentzer, Chang Liu, Beibei Wang, Guido C. Paesen, Jose Slon-Campos, César López-Camacho, Natasha M. Kafai, Adam L. Bailey, Rita E. Chen, Baoling Ying, Craig Thompson, Jai Bolton, Alex Fyfe, Sunetra Gupta, Tiong Kit Tan, Javier Gilbert-Jaramillo, William James, Michael Knight, Miles W. Carroll, Donal Skelly, Christina Dold, Yanchun Peng, Robert Levin, Tao Dong, Andrew J. Pollard, Julian C. Knight, Paul Klenerman, Nigel Temperton, David R. Hall, Mark A. Williams, Neil G. Paterson, Felicity K.R. Bertram, C. Alistair Siebert, Daniel K. Clare, Andrew Howe, Julika Radecke, Yun Song, Alain R. Townsend, Kuan-Ying A. Huang, Elizabeth E. Fry, Juthathip Mongkolsapaya, Michael S. Diamond, Jingshan Ren, David I. Stuart, and Gavin R. Screaton

Table S1. Summary of SARS-CoV-2-infected patients enrolled in the study, related to STAR Methods, Patient and blood samples.

**Participants**	
Female	16
Male	26
**Average Age (y)**	55.4 (IQR 47-61)
**Days post-symptom onset**	45.5 (IQR 40-53)
**Disease severity**	
Asymptomatic	1 (2.4%)
Mild	28 (66.6%)
Severe	12 (28.6%)
Critical	1 (2.4%)

Table S2. Neutralisation IC50 and biolayer interferometry (BLI) measurements of affinity (KD) and on (Ka) and off (Kdis) rates for selected mAbs (except for 159 BLI measurements are for Fabs). Related to STAR Methods Focus Reduction Neutralization Assay (FRNT) and Competition assay of antibodies.

IgG	Neutralization IC50 (ug/ml)#	KD (nM) $	Ka (1/Ms) $	Kdis (1/s) $
45	2.005*	5.9	7.8E04	4.6E-04
55	0.037 ± 0.008	3.6	1.1E05	3.9E-04
58	0.046 ± 0.016	3.4	1.0E05	3.6E-04
132	0.054 ± 0.014	3.1	1.1E05	3.3E-04
148	6.734*	4.0	7.6E04	3.0E-04
150	0.023 ± 0.003	0.9	2.1E05	1.9E-04
158	0.034 ± 0.003	0.7	1.2E05	8.4E-05
165	0.021 ± 0.006	4.8	7.0E04	3.3E-04
170	0.025*	2.7	1.7E05	4.5E-04
175	0.033 ± 0.009	2.4	2.8E05	6.5E-04
222	0.016 ± 0.003	3.0	1.2E05	3.5E-04
253	0.040 ± 0.007	4.5	1.4E05	6.1E-04
253H55L	0.004 ± 0.000	1.5	3.0E05	4.5E-04
253H165L	0.003 ± 0.000	3.1	1.2E05	3.9E-04
269	0.036 ± 0.009	2.2	1.8E05	3.9E-04
278	0.009 ± 0.002	3.0	2.6E05	7.8E-04
281	0.005 ± 0.001	1.0	2.3E05	2.3E-04
282	0.073 ± 0.009	1.5	1.6E05	2.4E-04
316	0.010 ± 0.002	2.0	3.5E05	7.1E-04
318	0.015 ± 0.002	7.6	6.7E04	5.1E-04
384	0.002 ± 0.001	2.7	1.1E05	2.9E-04
159 (anti-spike)	0.005 ± 0.001	1.1	5.7E05	6.3E-04

Neutralization activity of selected antibodies against SARS-CoV-2 were determined by FRNT. Data are from 3 independent experiments, each with duplicate wells and the data are shown as mean ± s.e.m.

*FRNT was performed once with duplicate wells.

$ Determined for Fab fragments

Table S3. Antibody coordinates, epitope assignment and IC50 values for the 80 RBD binders and pairs and triplets of non-competing antibodies from cluster analysis. Related to STAR Methods Competition mapping of antibodies. Coordinates in pseudo-water 'PDB' format: residue_ID=Ab_number, chain_ID=epitope, Occupancy=RMSD from Monte Carlo, B=Log(IC50[uM])

ATOM	1	AB	HOH	Z	1	219.988	209.996	175.029	7.43	2.00
ATOM	2	AB	HOH	W	2	241.900	217.482	139.154	8.51	2.00
ATOM	22	AB	HOH	Z	22	217.812	203.466	170.490	4.98	2.00
ATOM	38	AB	HOH	Z	38	219.118	209.290	156.724	12.77	2.00
ATOM	40	AB	HOH	V	40	227.132	202.980	138.890	0.00	-1.32
ATOM	45	AB	HOH	Y	45	243.041	201.662	165.392	5.90	0.92
ATOM	51	AB	HOH	Y	51	240.512	213.630	174.143	11.83	0.33
ATOM	54	AB	HOH	Z	54	224.162	216.829	178.651	10.51	2.00
ATOM	55	AB	HOH	V	55	230.392	202.791	136.613	6.47	-1.42
ATOM	57	AB	HOH	Y	57	244.237	208.839	171.111	17.86	0.25
ATOM	58	AB	HOH	X	58	248.948	211.008	154.261	3.28	-1.64
ATOM	61	AB	HOH	Z	61	215.370	201.510	158.862	9.19	-0.31
ATOM	75	AB	HOH	X	75	249.472	209.361	144.241	4.43	0.47
ATOM	78	AB	HOH	Y	78	243.439	212.348	167.679	8.02	2.00
ATOM	79	AB	HOH	Y	79	240.144	214.276	175.407	9.89	0.64
ATOM	88	AB	HOH	V	88	226.831	203.596	139.701	8.02	-1.51
ATOM	91	AB	HOH	Y	91	243.785	214.036	163.803	5.44	0.45
ATOM	120	AB	HOH	Y	120	241.052	215.135	166.628	8.48	2.00
ATOM	132	AB	HOH	V	132	225.151	203.146	140.780	7.34	-1.51
ATOM	140	AB	HOH	Z	140	218.037	201.529	170.470	7.17	2.00
ATOM	143	AB	HOH	Y	143	241.246	212.069	173.138	12.67	0.36
ATOM	144	AB	HOH	Y	144	239.212	219.880	163.947	9.32	0.53
ATOM	150	AB	HOH	V	150	225.177	199.643	138.903	6.21	-1.47
ATOM	155	AB	HOH	Z	155	218.779	195.978	165.617	10.90	0.88
ATOM	158	AB	HOH	V	158	225.142	200.374	139.122	6.14	-1.19
ATOM	163	AB	HOH	Z	163	216.360	203.294	166.704	7.82	0.91
ATOM	164	AB	HOH	X	164	240.165	218.613	161.087	5.45	2.00
ATOM	165	AB	HOH	V	165	229.346	202.859	137.309	8.73	-1.74
ATOM	170	AB	HOH	W	170	237.903	207.543	138.527	18.08	-0.32
ATOM	172	AB	HOH	Y	172	236.338	222.258	160.965	8.11	0.69
ATOM	175	AB	HOH	V	175	226.262	210.195	144.027	14.62	-1.66
ATOM	177	AB	HOH	Z	177	219.823	202.734	173.330	7.86	0.95
ATOM	178	AB	HOH	Z	178	217.146	205.246	158.026	11.23	0.46
ATOM	181	AB	HOH	W	181	237.022	211.814	140.007	8.31	-0.48
ATOM	182	AB	HOH	W	182	237.022	211.814	140.007	9.40	-0.68
ATOM	183	AB	HOH	W	183	234.883	216.679	140.697	11.84	-0.26
ATOM	185	AB	HOH	Y	185	240.359	217.188	164.380	10.53	2.00
ATOM	187	AB	HOH	W	187	236.113	215.662	140.482	9.30	0.29
ATOM	189	AB	HOH	Z	189	216.091	202.041	165.781	13.13	2.00
ATOM	217	AB	HOH	Y	217	241.885	215.173	164.589	6.05	0.85
ATOM	219	AB	HOH	V	219	231.498	192.382	152.478	25.53	-0.45
ATOM	222	AB	HOH	V	222	223.317	201.424	142.277	10.62	-1.66
ATOM	238	AB	HOH	W	238	235.903	214.120	140.733	13.95	13.95
ATOM	242	AB	HOH	W	242	239.488	211.308	138.735	7.00	7.00
ATOM	253	AB	HOH	V	253	229.489	200.810	134.904	11.11	-1.28
ATOM	263	AB	HOH	W	263	242.805	196.670	145.565	6.74	2.00
ATOM	264	AB	HOH	W	264	243.377	207.940	138.866	13.12	2.00
ATOM	269	AB	HOH	V	269	223.332	201.991	142.481	9.72	-1.28
ATOM	270	AB	HOH	W	270	242.360	195.917	146.938	9.16	1.00
ATOM	273	AB	HOH	Z	273	220.567	195.229	167.847	7.59	2.00
ATOM	278	AB	HOH	X	278	250.677	207.639	147.773	4.81	-1.23
ATOM	280	AB	HOH	Z	280	220.096	200.424	173.531	7.78	1.00
ATOM	281	AB	HOH	W	281	243.808	202.220	141.718	12.48	-2.22
ATOM	282	AB	HOH	V	282	224.323	198.309	139.537	4.46	-1.47
ATOM	292	AB	HOH	Y	292	244.625	207.249	171.203	9.89	0.68
ATOM	293	AB	HOH	Y	293	241.351	215.759	163.963	7.17	2.00
ATOM	300	AB	HOH	V	300	228.706	214.209	143.978	16.87	2.00
ATOM	301	AB	HOH	V	301	223.057	205.429	145.036	16.69	2.00
ATOM	314	AB	HOH	Z	314	220.665	209.331	165.719	9.16	2.00
ATOM	315	AB	HOH	X	315	250.105	211.317	149.429	3.55	0.27
ATOM	316	AB	HOH	W	316	236.386	202.405	135.598	13.71	-1.72
ATOM	318	AB	HOH	X	318	251.029	206.311	152.504	4.57	-0.33
ATOM	334	AB	HOH	Y	334	231.527	218.600	158.544	15.81	-0.24
ATOM	336	AB	HOH	Y	336	243.656	201.501	170.761	8.98	2.00
ATOM	337	AB	HOH	X	337	246.808	214.372	157.360	7.31	2.00
ATOM	340	AB	HOH	Z	340	218.843	197.397	169.870	11.79	2.00
ATOM	361	AB	HOH	X	361	250.487	206.197	150.651	5.41	-0.09
ATOM	369	AB	HOH	Y	369	243.446	201.373	166.570	14.97	0.01
ATOM	373	AB	HOH	Z	373	230.940	192.660	163.268	9.00	2.00
ATOM	379	AB	HOH	X	379	246.540	202.168	150.446	9.74	1.00
ATOM	382	AB	HOH	X	382	246.528	202.685	156.748	4.09	-0.20
ATOM	384	AB	HOH	W	384	240.568	201.666	137.825	8.34	-2.40
ATOM	386	AB	HOH	X	386	243.678	201.814	160.033	6.14	2.00
ATOM	387	AB	HOH	Y	387	242.323	201.191	165.021	10.29	2.00
ATOM	393	AB	HOH	Z	393	229.130	191.944	161.721	9.73	2.00
ATOM	394	AB	HOH	W	394	242.360	195.917	146.938	16.59	-1.77
ATOM	398	AB	HOH	W	398	239.534	192.608	143.991	13.34	-1.07
ATOM	400	AB	HOH	Y	400	242.184	200.410	166.818	13.16	2.00
ATOM	411	AB	HOH	W	411	231.577	214.255	143.535	16.41	0.03
ATOM	412	AB	HOH	W	412	235.358	213.955	140.720	16.23	1.00

max 20% competition <0.1 ug/ml IC50

55	58
55	278
58	88
58	132
58	150
58	158
58	165
58	175
58	222
58	282
88	278
132	278
150	278
158	278
165	278
175	278
222	278
278	282

max 20% competition <1 ug/ml IC50

40	318	
40	334	
40	382	
55	58	61
55	61	278
55	61	318
55	61	334
55	61	361
55	61	382
58	61	165
58	61	175
58	88	
58	132	
58	150	
58	158	
58	222	
58	282	
61	165	278
61	165	318
61	165	334
61	165	361
61	165	382
61	170	334
61	175	278
61	175	318
61	175	334
61	175	361
61	175	382
61	181	334
61	182	334
61	183	334
61	281	334
61	316	334
61	334	384
61	334	394
61	334	398
88	278	
88	318	
88	334	
88	361	
88	382	
132	278	
132	318	
132	334	
132	361	
132	382	
150	278	
150	318	
150	334	
150	361	
150	382	
158	278	
158	318	
158	334	

Table S4. Stability tests for selected antibodies using thermofluor and dynamic light scattering (DLS). Related to STAR Methods Physical assays.

	Thermofluor (reporter dye)				DLS @ 20 C		Fraction soluble after N freeze-thaw cycles	
Antibody id	Tm 1	Prop^n of melt	Tm 2	Prop^n of melt	Peak1 MW	Peak1 %mass	N=5	N=20
2	78.4				146.25	100		
22	80.7				177.55	99.98		
40	73.8				215.20	100.00	0.9	0.8
44	77.6	0.9	89.1	0.1	685.57*	100.00		
45	75.9	0.3	86.6	0.8	146.17	100.00	1.0	0.8
54	71.9	0.9	87.2	0.1	177.31	99.71		
55	75.9	0.4	86.7	0.7	215.20	99.83	1.0	0.9
58	80.6				213.93	99.98	0.9	0.9
61	75.9	0.9	87.1	0.1	146.17	100.00		
75	75.3	0.4	84.1	0.7	215.08	100.00	0.9	1.0
88	73.3				213.93	100.00	1.0	0.9
111	80.1				214.23	100.00		
132	69.1	0.7	79.7	0.3	146.25	99.96	1.0	0.6
140	79.0				260.07	99.90		
148	75.4	0.3	85.4	0.7	213.93	99.98		
150	71.1	0.9	89.1	0.1	176.08	100.00	1.0	0.7
158	76.4	0.9	88.4	0.1	213.93	99.91	1.0	0.9
165	78.2				177.31	100.00	1.0	1.0
170	64.9	0.8	76.1	0.2	Out of Range	99.16	0.9	1.0
175	74.3				215.08	100.00	1.0	0.9
177	74.3				176.08	99.93		
222	76.7				214.05	100.00	0.9	0.9
253	75.9				146.17	100.00	1.0	0.9
269	73.7				214.90	100.00	0.9	0.8
278	76.2	1.0	88.0	0.1	316.65	100.00	1.0	0.8
281	70.5				214.23	100.00	0.9	0.8
282	70.8	0.9	88.3	0.1	215.08	99.99	0.9	0.9
316	74.9				465.66	100.00	0.9	0.9
318	72.1	0.9	86.7	0.1	144.84	100.00		
384	74.9	0.9	88.0	0.1	146.17	100.00	1.0	0.8
253H165L	74.5	0.6	86.4	0.4	176.08	100.00	0.9	0.7
253H55L	74.2	0.6	87.3	0.4	175.84	100.00	0.9	0.9

Table S5. X-ray data collection and refinement statistics (molecular replacement). Related to STAR Methods X-ray data collection, structure determination and refinement.

**Structure**	RBD-88-45	RBD-150	RBD-158 (form 1)	RBD-158 (form 2)	RBD-253-75	RBD-253H55L-75	RBD-scFV269	RBD-316	RBD-384-S309
PDB ID	7BEL	7BEI	7BEJ	7BEK	7BEN	7BEO	7BEM	7BEH	7BEP
**Data collection**									
Space group	$C2$[a]	$C222_1$	$C222_1$	$C222_1$	$P2_12_12_1$[a]	$P2_1$	$P6_222$	$P2_12_12$	$P2_12_12_1$
Cell dimensions									
a, b, c (Å)	180.1, 140.7, 131.0	81.4, 150.7, 145.5	53.2, 232.3, 135.2	83.0, 149.4, 145.5	93.2, 149.8, 229.1	93.4, 150.1, 116.1	173.6, 173.6, 120.6	104.0, 150.9, 46.0	108.8, 113.2, 302.8
α, β, γ (°)	90, 124.6, 90	90, 90, 90	90, 90, 90	90, 90, 90	90, 90, 90	90, 92.0, 90	90, 90, 120	90, 90, 90	90, 90, 90
Resolution (Å)	59–2.53 (2.57–2.53)[b]	52–2.30 (2.34–2.30)	53–2.42 (2.46–2.42)	51–2.04 (2.08–2.04)	59–2.50 (2.54–2.50)	66–3.19 (3.25–3.19)	57–2.52 (2.56–2.52)	52–2.30 (2.34–2.30)	63–2.61 (2.65–2.61)
R_{merge}	0.189 (---)	0.192 (0.990)	0.250 (---)	0.118 (---)	0.283 (---)	0.412 (---)	0.432 (---)	0.107 (---)	0.112 (---)
R_{pim}	0.079 (1.101)	0.039 (0.362)	0.051 (0.781)	0.033 (1.296)	0.056 (1.526)	0.169 (1.189)	0.051 (2.921)	0.032 (1.042)	0.032 (0.982)
$I/\sigma(I)$	5.6 (0.3)	12.5 (0.5)	7.6 (0.4)	13.1 (0.5)	7.3 (0.3)	4.0 (0.5)	9.2 (0.2)	14.2 (0.6)	13.1 (0.5)
$CC_{1/2}$	0.995 (0.326)	0.997 (0.651)	0.993 (0.363)	0.999 (0.345)	0.999 (0.623)	0.977 (0.280)	0.999 (0.627)	0.998 (0.223)	0.998 (0.344)
Completeness (%)	97.8 (75.6)	84.4 (42.8)	98.5 (85.2)	99.9 (97.2)	100 (99.5)	100 (99.5)	99.9 (96.3)	85.7 (40.2)	100 (99.1)
Redundancy	6.6 (5.0)	22.0 (8.2)	23.7 (13.5)	13.4 (9.9)	26.6 (24.8)	6.9 (7.1)	73.4 (54.1)	11.0 (4.9)	13.3 (12.3)
**Refinement**									
Resolution (Å)	49–2.53	43–2.30	53–2.42	43–2.04	58–2.50	58–3.19	47–2.52	45–2.30	63–2.61
No. reflections	81142/4196	30799/1567	30107/1610	54733/2897	105179/5570	50411/2657	33910/1753	26846/1392	108562/5577
R_{work} / R_{free}	0.250/0.285	0.197/0.236	0.195/0.229	0.200/0.220	0.242/0.284	0.232/0.274	0.217/0.248	0.218/0.233	0.206/0.241
No. atoms									
Protein	13221	4787	4777	4816	16052	16152	3235	4768	16682
Ligand/ion	158	47	68	98	187	177	47	104	234
B factors (Å^2)									
Protein	70	49	58	63	73	90	91	60	98
Ligand/ion	86	84	82	91	97	114	139	74	124
r.m.s. deviations									
Bond lengths (Å)	0.003	0.005	0.003	0.002	0.002	0.002	0.003	0.002	0.003
Bond angles (°)	0.7	0.8	0.6	0.6	0.6	0.5	0.6	0.5	0.6

[a]Presence of translational NCS. [b]Values in parentheses are for highest-resolution shell.

Table S6. Cryo-EM data collection, refinement and validation statistics for Spike/Fab(IgG) complexes.
Related to STAR Methods Cryo-EM Data collection and processing.

Complex (EMD ID, PDB ID)	**40** (EMD-12274, 7ND3)	**88** (EMD-12275, 7ND4)	**150** (EMD-12276, 7ND5)	**158*** (EMD-12277, 7ND6)	**316** (EMD-12278, 7ND7)	**384** (EMD-12279, 7ND8)	**253H55L** (EMD-12280, 7ND9)	**253H55L** (EMD-12281, 7NDA)	**253H165L** (EMD-12282, 7NDB)	**159** (EMD-12283, 7NDC)	**159** (EMD-12284, 7NDD)
**Data collection and processing**											
Voltage (kV)					300						
Electron exposure (e⁻/Å^2)	43.1	57.6	48.8	45.3	46.0	47.7	47.2	47.2	44.7	50.5	50.5
Defocus range (μm)					-0.8 to -2.6						
Pixel size [super res] (Å)	0.83 [0.415]	0.82	0.82	0.82	0.82	0.82	0.83 [0.415]	0.83 [0.415]	0.83 [0.415]	0.82	0.82
Symmetry imposed	*C1*	*C3*	*C1*	*C1*	*C3*	*C1*	*C1*	*C1*	*C1*	*C3*	*C1*
Particles in final reconstruction (no.)	39186	56686	93555	18768	162,905	73,158	206,548	47,242	31,477	13638	27880
Map resolution (Å)	3.7	3.1	3.4	6.8	3.6	3.5	2.8	3.3	4.6	4.1	4.2
FSC threshold	0.143	0.143	0.143	0.143	0.143	0.143	0.143	0.143	0.143	0.143	0.143
**Refinement**											
Initial model used	PDB 6Z97	PDB 6ZDH	PDB 6Z97	PDB 6Z97	PDB 7NDA	PDB 7NDA	PDB 6Z97	PDB 6VXX	PDB 6Z97	PDB 7NDA	PDB 7ND5
RBD conformation	One-up	All-up	One-up	One-up	All-down	All-down	One-up	All-down	One-up	All-down	One-up
Model resolution (Masked) (Å)	3.8	3.2	3.5	7.6	3.8	3.8	3.0	3.5	4.9	4.2	4.2
FSC threshold	0.5	0.5	0.5	0.5	0.5	0.5	0.5	0.5	0.5	0.5	0.5
Map sharpening *B* factor (Å^2)	-83	-56	-45	-369	-90	-48	-61	-37	-133	-61	-49
Model composition											
Non-hydrogen atoms	25050	29848	26710	25152	29591	27659	27336	27729	27184	30648	30645
Protein residues	3194	3727	3406	26742	3699	3447	3482	3452	3460	3852	3852
Ligands	49	71	49	49	64	60	50	63	49	54	54
B factors (Å^2)											
Protein	136	78	110	396	87	147	82	126	264	184	239
Ligand	135	89	96	402	104	149	94	137	258	169	183
R.m.s. deviations											
Bond lengths (Å)	0.002	0.003	0.002	0.003	0.003	0.004	0.002	0.003	0.002	0.002	0.003
Bond angles (°)	0.5	0.6	0.5	0.6	0.6	0.7	0.5	0.6	0.5	0.5	0.6
**Validation**											
MolProbity score	1.40	1.47	1.45	1.43	1.41	1.44	1.56	1.61	1.58	1.44	1.54
Clashscore	3.47	3.79	4.18	3.98	3.57	4.05	4.31	5.27	4.93	3.82	4.67
Poor rotamers (%)	0.64	0.28	0.32	0.53	0.38	0.30	0.62	0.30	0.72	0.37	0.40
Ramachandran plot											
Favored (%)	96.2	95.6	96.3	96.4	96.2	96.3	94.9	95.3	95.3	96.0	95.7
Allowed (%)	3.7	4.1	3.7	3.6	3.2	3.5	4.9	4.4	4.5	4.0	4.1
Disallowed (%)	0.1	0.3	0	0	0.2	0.2	0.2	0.3	0.2	0	0.2

*, Rigid body refinement only.

Table S7. Affinity (KD), neutralization potency (IC50) and % occupancy against SARS-CoV-2 of full-length IgG and Fab for ten selected antibodies were determined. Related to STAR Methods Focus Reduction Neutralization Assay (FRNT) and Competition assay of antibodies.
Data are from 2 independent experiments, each with duplicate wells and the data are shown as mean ± s.e.m. .

Antibody	Binding KD (nM)	Neutralization IC50 (nM)	%Occupancy
40 IgG	0.33 ± 0.04	0.16 ± 0.03	33.3 ± 1.3
40 Fab	1.23 ± 0.19	45.68 ± 7.15	97.4 ± 0.0
88 IgG	0.25 ± 0.01	0.07 ± 0.04	21.9 ± 9.5
88 Fab	1.21 ± 0.14	6.95 ± 0.07	85.2 ± 1.6
150 IgG	0.24 ± 0.01	0.08 ± 0.02	25.1 ± 5.7
150 Fab	0.75 ± 0.10	0.61 ±0.23	43.2 ± 6.6
158 IgG	0.50 ± 0.02	0.13 ± 0.02	20.5 ± 2.6
158 Fab	3.73 ± 0.56	4.12 ± 0.04	52.7 ± 3.5
159 IgG	0.19 ± 0.02	0.08 ± 0.01	30.0 ± 5.2
159 Fab	0.29 ± 0.02	N/A	N/A
253 IgG	0.17 ± 0.01	0.36 ± 0.02	67.9 ± 2.3
253 Fab	23.38 ± 3.84	N/A	N/A
253H55L IgG	0.09 ± 0.01	0.02 ± 0.00	19.6 ± 2.1
253H55L Fab	10.19 ± 0.56	5.08 ± 0.47	33.2 ± 0.8
253H165L IgG	0.09 ± 0.01	0.03 ± 0.00	24.5 ± 0.6
253H165L Fab	5.02 ± 1.40	5.22 ± 1.18	51.3 ± 1.4
316 IgG	0.13 ± 0.00	0.08 ± 0.02	35.4 ± 6.0
316 Fab	8.76 ± 0.64	27.64 ± 9.47	74.0 ± 8.1
384 IgG	0.10 ± 0.03	0.01 ± 0.00	12.1 ± 2.3
384 Fab	7.86 ± 0.96	4.86 ± 0.16	38.4 ± 3.7
